# Proteome profiling reveals novel biomarkers to identify complicated parapneumonic effusions

**DOI:** 10.1038/s41598-017-04189-4

**Published:** 2017-06-22

**Authors:** Kuo-An Wu, Chih-Ching Wu, Chi-De Chen, Chi-Ming Chu, Li-Jane Shih, Yu-Ching Liu, Chih-Liang Wang, Hsi-Hsien Lin, Chia-Yu Yang

**Affiliations:** 1 Graduate Institute of Clinical Medical Sciences, College of Medicine, Chang Gung University, Taoyuan, Taiwan; 2 Department of Internal Medicine, Taoyuan Armed Forces General Hospital, Taoyuan, Taiwan; 3 Department of Medical Biotechnology and Laboratory Science, College of Medicine, Chang Gung University, Taoyuan, Taiwan; 4 Department of Otolaryngology-Head & Neck Surgery, Chang Gung Memorial Hospital, Taoyuan, Taiwan; 5 Molecular Medicine Research Center, Chang Gung University, Taoyuan, Taiwan; 6 Division of Biomedical Statistics and Informatics, School of Public Health, National Defense Medical Center, Taipei, Taiwan; 7 Department of Medical Laboratory, Taoyuan Armed Forces General Hospital, Taoyuan, Taiwan; 8 Graduate Institute of Medical Sciences, National Defense Medical Center, Taipei, Taiwan; 9 Division of Pulmonary Oncology and Interventional Bronchoscopy, Department of Thoracic Medicine, Chang Gung Memorial Hospital, Taoyuan, Taiwan; 10 Department of Microbiology and Immunology, College of Medicine, Chang Gung University, Taoyuan, Taiwan; 11 Chang Gung Immunology Consortium and Department of Anatomic Pathology, Chang Gung Memorial Hospital, Taoyuan, Taiwan; 12 Division of Colon and Rectal Surgery, Department of Surgery, Chang Gung Memorial Hospital, Taoyuan, Taiwan

## Abstract

Patients with pneumonia and parapneumonic effusion (PPE) have elevated mortality and a poor prognosis. The aim of this study was to discover novel biomarkers to help distinguish between uncomplicated PPE (UPPE) and complicated PPE (CPPE). Using an iTRAQ-based quantitative proteomics, we identified 766 proteins in pleural effusions from PPE patients. In total, 45 of these proteins were quantified as upregulated proteins in CPPE. Four novel upregulated candidates (BPI, NGAL, AZU1, and calprotectin) were selected and further verified using enzyme-linked immunosorbent assays (ELISAs) on 220 patients with pleural effusions due to different causes. The pleural fluid levels of BPI, NGAL, AZU1, and calprotectin were significantly elevated in patients with CPPE. Among these four biomarkers, BPI had the best diagnostic value for CPPE, with an AUC value of 0.966, a sensitivity of 97%, and a specificity of 91.4%. A logistic regression analysis demonstrated a strong association between BPI levels > 10 ng/ml and CPPE (odds ratio = 341.3). Furthermore, the combination of pleural fluid BPI levels with LDH levels improved the sensitivity and specificity to 100% and 91.4%, respectively. Thus, our findings provided a comprehensive effusion proteome data set for PPE biomarker discovery and revealed novel biomarkers for the diagnosis of CPPE.

## Introduction

A parapneumonic effusion (PPE) is an accumulation of exudative pleural fluid that occurs in association with an ipsilateral pulmonary infection. PPEs are present in 20% to 40% of hospitalized patients with pneumonia^1^. Based on fluid characteristics and pathogenesis, PPEs are classified into the following three groups: uncomplicated PPE (UPPE), complicated PPE (CPPE), and thoracic empyema^2^. The occurrence of CPPE or empyema greatly increases the risk of morbidity and mortality compared to that of UPPE^1^. Generally, UPPE can be cured with antibiotic treatment alone. When CPPE and empyema are present, pleural space drainage is mandatory^2^. For conditions such as patients with loculated pleural collection or poor clinical progress during treatment with antibiotics alone, drainage treatment will also be required^3^. Thus, an accurate diagnosis in the early stages of PPE is important for clinicians.

The classical criteria for clinical diagnosis of PPE is based on the biochemical parameters of the pleural effusions, including levels of lactate dehydrogenase (LDH), glucose, and pH^1^. To improve PPE diagnosis, numerous biomarkers, such as inflammatory cytokines (tumour necrosis factor-alpha/TNF-α, interleukin-8/IL-8, and IL-1β), enzymes (neutrophil elastase, myeloperoxidase/MPO, and metalloproteinases/MMPs), C-reactive protein (CRP), and soluble triggering receptor expressed on myeloid cells (sTREM-1), have been evaluated^4–9^. In these reports, the candidates were selected by a literature search and confirmed by immunoassays^10^.

Currently, high-throughput proteomics technology provides more comprehensive proteome profiling of body fluids, which facilitates biomarker discovery^11–13^. Thus, the characterization of proteomic changes associated with PPE progression helps to elucidate disease mechanisms and identify useful biomarkers and therapeutic targets. In this study, we aimed to investigate useful biomarkers for the differential diagnosis of UPPE and CPPE, with the goal of identifying specific proteins and pathways important for the molecular mechanisms of PPE progression. Using the comprehensive proteomics approach, we generated the PPE proteome data set for biomarker research and verified the levels of four novel proteins (BPI, NGAL, AZU1, and calprotectin) in PPE. Collectively, we identified novel biomarkers for the diagnosis of CPPE.

## Results

### Proteome profiling of UPPE and CPPE by iTRAQ-based mass spectrometry

**Figure 1:**
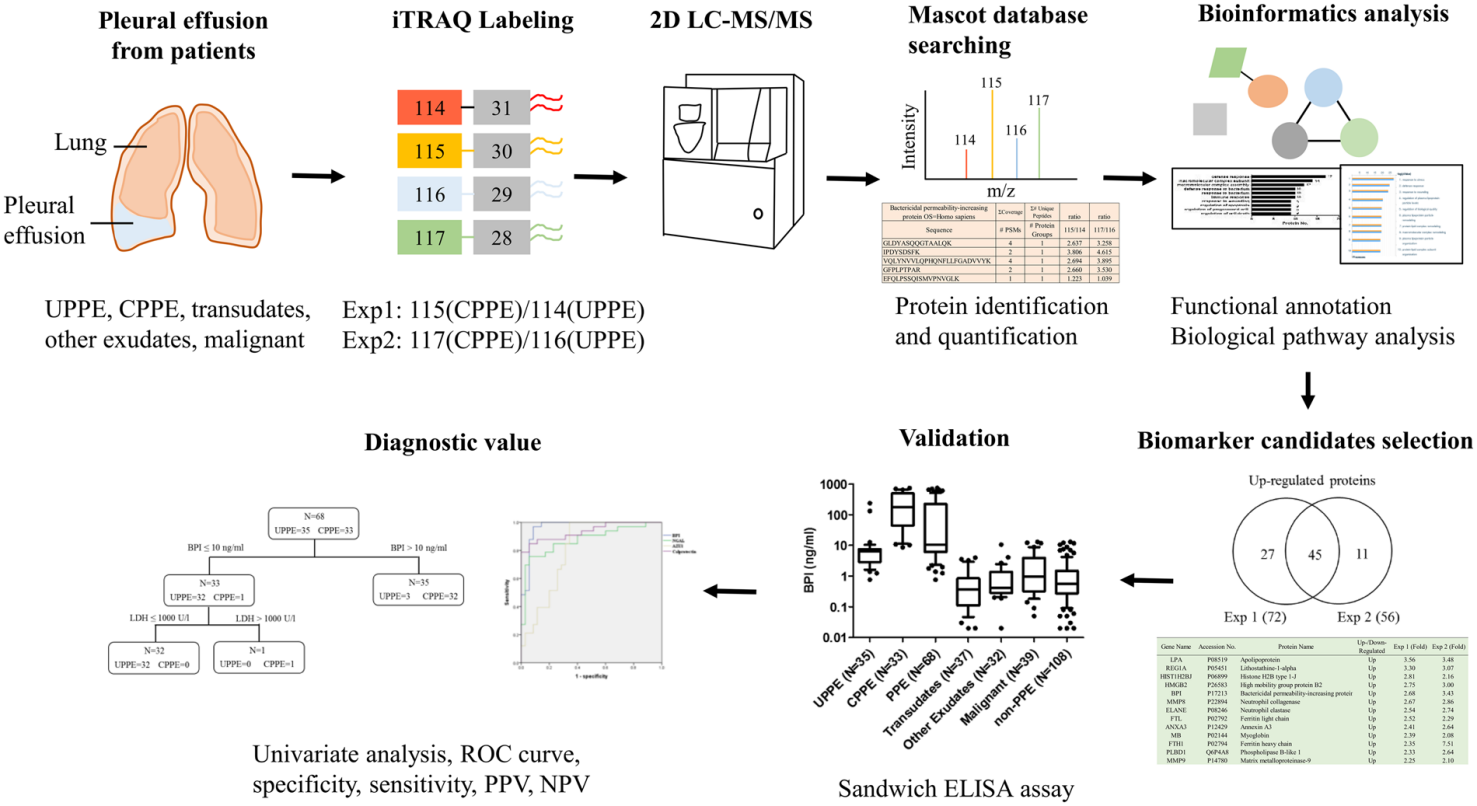
Schematic diagram of the experimental design for identifying novel biomarkers in parapneumonic effusions. Pleural effusions were collected from patients with different causes, including transudates, other exudates, malignant, uncomplicated parapneumonic effusion (UPPE) and complicated parapneumonic effusion (CPPE). Four samples from each type of UPPE or CPPE were pooled, processed, and labeled with iTRAQ reagents, following by 2D LC-MS/MS analysis. Peptide and protein were identified and quantified using MASCOT software. Selected proteins were uploaded into the DAVID database and MetaCore software to evaluate possible associations with known pathways and biological processes. The potential newer biomarkers were validated in an extended patients group by ELISA assays. Statistical analysis was performed to determine the efficacy of biomarkers in discriminating between UPPE and CPPE patients.

To identify novel biomarkers for PPE diagnosis, the proteomes of pleural effusions from patients with UPPE and CPPE (Supplementary Table 1) were analyzed using iTRAQ-based mass spectrometry (Fig. 1). After depletion of six abundant proteins (albumin, immunoglobulin G, immunoglobulin A, transferrin, α1-antitrypsin, and haptoglobin) using affinity column, the pleural effusion samples from 4 UPPE patients and 4 CPPE patients were pooled equally (10 μg proteins from each patient) into the UPPE group and the CPPE group (Exp 1), respectively, to diminish the influences of between-individual variations. For technical replicates, the second sets of samples from UPPE patients and CPPE patients were prepared from different batches (Exp 2). The two set of samples were digested with trypsin and then labeled with the iTRAQ reagent in parallel. As shown in Fig. 1, iTRAQ 114 and 116 reagents were combined with peptides from UPPE samples of Exp 1 and Exp 2, respectively. Peptides from CPPE samples of Exp 1 and Exp 2 were labeled with iTRAQ 115 and 117 reagents, respectively. The iTRAQ-labeled samples were then analyzed by two-dimensional LC-MS/MS for quantitative proteomic analysis. The two-dimensional fractionation of the labeled peptides involved the use of an online SCX separation in the first dimension, followed by an online reverse phase fractionation. Each fraction was analyzed using a LTQ-Orbitrap system. The MS/MS spectra were searched against the Swiss-Prot human sequence database with the Mascot algorithm in the Proteome Discoverer software. The search results were further filtered with high confidence of peptide identification and at least two peptide hits for each protein identification to ensure an overall false-discovery rate below 0.01. Using this approach, we identified 766 non-redundant proteins and quantified 740 and 738 proteins in Exp 1 and Exp 2, respectively (Supplementary Fig. 1A). Proteins with CPPE/UPPE ratios below the mean of all ratios minus the standard deviation (SD) of all ratios were considered to be underexpressed (0.658 and 0.668 for Exp 1 and Exp 2, respectively). Proteins above the mean plus the SD were considered to be overexpressed (1.381 and 1.593 for Exp 1 and Exp 2, respectively). Only those proteins with altered expression in both experiments were considered potential candidates for dysregulated proteins in the CPPE group. This approach increases the probability of discovering candidates that are affected by PPE progression irrespective of experimental variations. Using these criteria, 80 proteins were identified with differential expression in both Exp 1 and Exp 2. Among them, 45 proteins with higher expression levels and 35 proteins with lower expression levels in the CPPE group were found in both experimental replicates (Supplementary Fig. 1B). The detail information of these 45 up-regulated proteins and 35 down-regulated proteins in CPPE were summarized in Supplementary Table 2 and Supplementary Table 3, respectively.

### Pathway analysis of 45 proteins up-regulated in CPPE compared with UPPE

To explore the biological significances of these 45 proteins up-regulated in CPPE compared with UPPE in depth, the biological processes, pathway annotations, and molecular functions were revealed using DAVID Gene Functional Classification Tool (v6.7). The enrichment biological processes revealed that the up-regulated proteins were significantly involved in the defense response, immune response, macromolecular complex subunit organization, and defense response to bacteria (Table 1). Moreover, the differentially expressed proteins were analyzed with MetaCore bioinformatics software to determine the GO of cellular processes involved in CPPE and UPPE. The top three most significant cellular processes were those related to responses to bacteria, defense responses to bacteria, and nucleosome assembly (Supplementary Table 4). These results collectively indicated that proteins involved in the defense response to bacteria and immune response were significantly associated with CPPE.

**Table 1:** Enrichment analysis of biological processes for up-regulated proteins in effusions.

Biological process	Count	Identified proteins involved in the process	*p* value
Defense response	16	CRISP3, RNASE3, S100A8, S100A9, LYZ, ANXA1, PGLYRP1, CD5L, MIF, AZU1, BPI, HIST1H2BK, HIST1H2BJ, MPO, DEFA1, CTSG	1.95 × 10^−10^
Immune response	10	CRISP3, BPI, IGHD, PGLYRP1, IGLL1, DEFA1, FCGR3A, CTSG, FTH1, MIF	2.17 × 10^−4^
Macromolecular complex subunit organization	10	VWF, HMGB2, VCP, HIST1H2BK, HIST1H4A, HIST1H2BJ, HIST1H2AH, MPO, ADIPOQ, MIF	2.68 × 10^−4^
Defense response to bacterium	9	AZU1, BPI, RNASE3, HIST1H2BK, HIST1H2BJ, PGLYRP1, LYZ, DEFA1, CTSG	1.60 × 10^−9^
Response to wounding	8	AZU1, VWF, LPA, S100A8, S100A9, ANXA1, LYZ, MIF	1.11 × 10^−3^
Inflammatory response	6	AZU1, S100A8, S100A9, ANXA1, LYZ, MIF	3.18 × 10^−3^
Nucleosome assembly	5	HMGB2, HIST1H2BK, HIST1H4A, HIST1H2BJ, HIST1H2AH	1.29 × 10^−4^

DAVID (version 6.7) was applied to functionally annotate enriched proteins, using the annotation category GOTERM_BP_FAT. Processes with at least five protein members and *p* values less than 0.01 were considered significant.

### Elevated pleural fluid levels of BPI, NGAL, AZU1, and calprotectin in PPE, particularly individuals with CPPE

**Figure 2:**
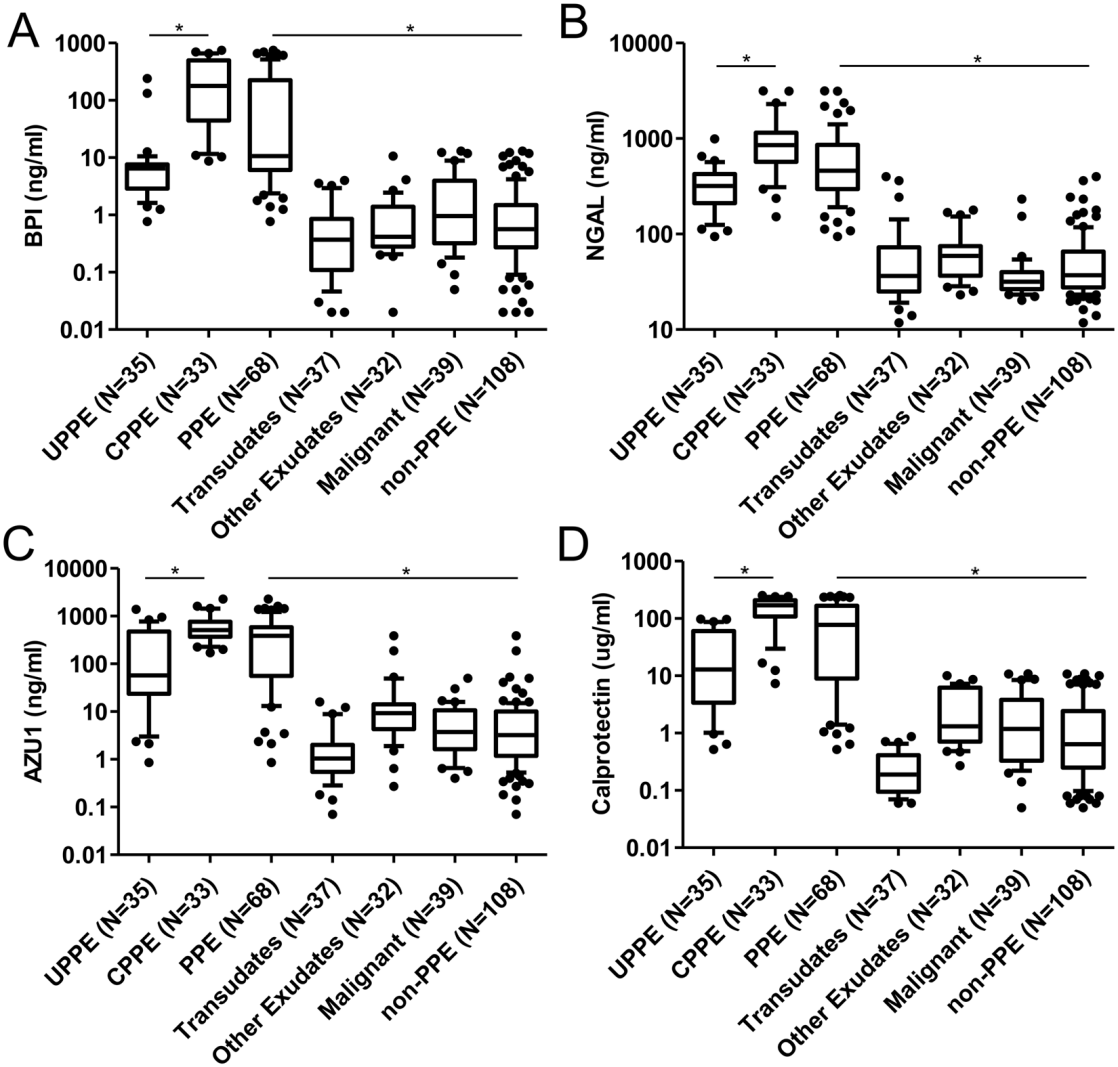
Box plots of the concentrations of four biomarkers in five types of pleural effusions. The pleural effusion levels of (**A**) bactericidal permeability-increasing protein (BPI), (**B**) neutrophil gelatinase-associated lipocalin (NGAL), (**C**) azurocidin (AZU1), and (**D**) calprotectin from patients with UPPE, CPPE, transudates, other exudates, and malignant were determined by sandwich ELISA. PPE refer to UPPE and CPPE. non-PPE refer to transudates, other exudates, and malignant. Horizontal lines represent mean values. *P < 0.0001, indicates statistical significance using nonparametric Mann-Whitney U test. PPE: parapneumonic effusion; UPPE: uncomplicated parapneumonic effusion; CPPE: complicated parapneumonic effusion.

According to the proteomics data and bioinformatics analysis, four proteins were selected as potential biomarker candidates, including BPI, NGAL, AZU1, and calprotectin, and their roles in pleural effusions have not been addressed before. These proteins were further verified by sandwich ELISA. We determined the pleural fluid levels of these four candidates in 176 patients with five different causes of pleural effusion (Table 2; PPE refers to UPPE and CPPE; non-PPE refers to transudates, other exudates, and malignant effusions). As shown in Fig. 2, the pleural fluid levels of BPI, NGAL, AZU1, and calprotectin in PPEs were significantly higher than those in non-PPEs. Among the five types of pleural effusions, the protein levels of BPI, NGAL, AZU1, and calprotectin in CPPE were highest and estimated (expressed as the mean values ± s.e.m.) as 274.5 ± 43.6 ng/ml, 1035.5 ± 130.4 ng/ml, 669.5 ± 84.5 ng/ml, and 152.7 ± 12.1 μg/ml, respectively (Table 3). The true positive rate (sensitivity) was plotted against the false positive rate (100% − specificity), and the area under the curve (AUC) values were reported with a 95% confidence interval as an estimate of diagnostic usefulness. The AUCs for distinguishing PPE from non-PPE were 0.946 for BPI, 0.983 for NGAL, 0.943 for AZU1, and 0.931 for calprotectin (Table 4).

**Table 2:** Demographics and pleural fluid data of the study population.

Characteristics	PPE			non-PPE		
	UPPE	CPPE	*p *value	Transudates	Other exudates	Malignant
Patients	35	33	—	37	32	39
Male (%)	28 (80%)	29 (87.9%)	—	24 (64.9%)	21 (65.6%)	17 (43.6%)
Age (years)^a^	69.75 ± 3.12	66.75 ± 3.01	0.585^b^	75.48 ± 2.65	75.31 ± 3.30	67.73 ± 1.67
Proteins (g/dl)^a^	3.64 ± 0.14	4.06 ± 0.21	0.029^b^	1.78 ± 0.11	3.58 ± 0.19	4.41 ± 0.14
Glucose (mg/dl)^a^	154.67 ± 13.09	45.23 ± 9.05	< 0.001^b^	160.58 ± 7.12	142.59 ± 9.85	118.38 ± 17.29
LDH (U/l)^a^	401.14 ± 52.05	5781.88 ± 1744.09	< 0.001^b^	82.40 ± 4.37	231.93 ± 29.70	615.97 ± 131.00
pH^a^	7.45 ± 0.02	7.00 ± 0.06	< 0.001^b^	7.46 ± 0.01	7.44 ± 0.09	N.A.^c^

^a^Data are presented as mean ± s.e.m.

^b^The *p* value of Mann-Whitney U test presents the difference between uncomplicated parapneumonic effusion (UPPE) and complicated parapneumonic effusion (CPPE).

^c^N.A. means data not available.

**Table 3:** Pleural fluid levels of new biomarkers in five types of pleural effusions.

First cohort (n = 176)									
Biomarker candidates	UPPE (n = 35)	CPPE (n = 33)	*p *value	PPE (n = 68)	Transudates (n = 37)	Other exudates (n = 32)	Malignant (n = 39)	non-PPE (n = 108)	*p *value
BPI (ng/ml)^a^	15.9 ± 7.5	274.5 ± 43.6	< 0.001^b^	141.4 ± 26.5	0.8 ± 0.2	1.1 ± 0.3	2.7 ± 0.6	1.6 ± 0.3	< 0.001^c^
NGAL (ng/ml)^a^	341.4 ± 30.9	1035.5 ± 132.4	< 0.001^b^	678.2 ± 78.2	71.7 ± 14.3	67.6 ± 7.3	41.8 ± 6.1	59.7 ± 5.9	< 0.001^c^
AZU1 (ng/ml)^a^	248.3 ± 56.4	669.5 ± 84.5	< 0.001^b^	452.7 ± 56.1	2.4 ± 0.6	28.4 ± 13.0	7.3 ± 1.5	11.9 ± 4.0	< 0.001^c^
Calprotectin (μg/ml)^a^	29.8 ± 5.7	152.7 ± 12.1	< 0.001^b^	89.4 ± 9.9	0.3 ± 0.03	9.7 ± 6.6	2.6 ± 0.5	3.9 ± 2.0	< 0.001^c^
**Validation cohort (n = 44)**									
**Biomarker candidates**	**UPPE (n** = **26)**	**CPPE (n** = **18)**	***p *** **value**	—	—	—	—	—	—
BPI (ng/ml)^a^	11.4 ± 7.9	222.3 ± 46.1	< 0.001^b^	—	—	—	—	—	—
NGAL (ng/ml)^a^	240.4 ± 74.1	1416.2 ± 272.6	< 0.001^b^	—	—	—	—	—	—
AZU1 (ng/ml)^a^	205.4 ± 47.2	753.7 ± 117.8	< 0.001^b^	—	—	—	—	—	—
Calprotectin (μg/ml)^a^	9.2 ± 1.9	118.2 ± 16.1	< 0.001^b^	—	—	—	—	—	—

^a^Data are presented as mean ± s.e.m.

^b^The *p* value of Mann-Whitney U test presents the difference between uncomplicated parapneumonic effusion (UPPE) and complicated parapneumonic effusion (CPPE).

^c^The *p* value of Mann-Whitney U test presents the difference between parapneumonic effusion (PPE, refer to UPPE and CPPE) and non parapneumonic effusion (non-PPE, refer to transudates, other exudates, and malignant) in this study.

**Table 4:** Operating characteristics of newer pleural fluid tests for identifying PPE from non-PPE.

Biomarker candidates	Cutoff	Sensitivity (%)	Specificity (%)	PPV (%)	NPV (%)	AUC (95% confidence interval)
BPI	> 4.5 ng/ml	86.8	90.7	85.5	91.6	0.946 (0.916–0.976)
NGAL	> 169.9 ng/ml	92.6	95.4	92.6	95.4	0.983 (0.969–0.997)
AZU1	> 18.8 ng/ml	89.7	93.5	89.7	93.5	0.943 (0.903–0.982)
Calprotectin	> 11.6 μg/ml	73.5	99.1	98.0	85.6	0.931 (0.893–0.968)

PPV: positive predictive values; NPV: negative predictive values; AUC: area under ROC curve; BPI: bactericidal permeability-increasing protein; NGAL: neutrophil gelatinase-associated lipocalin; AZU1: azurocidin. The parapneumonic effusion (PPE, refer to UPPE and CPPE) and non-parapneumonic effusion (non-PPE, refer to transudates, other exudates, and malignant) in this study.

### Decision values of BPI, NGAL, AZU1, and calprotectin for distinguishing between UPPE and CPPE

We next evaluated the diagnostic performance of these four novel biomarkers and three traditional biochemical parameters (pH, Glucose, and LDH) in identifying CPPE. The AUCs for distinguishing CPPE from UPPE were 0.966 for BPI, 0.875 for NGAL, 0.807 for AZU1, 0.937 for calprotectin, 0.915 for LDH, 0.907 for glucose, and 0.947 for pH value. Of the biomarkers studied, BPI had the best diagnostic value for CPPE, with a sensitivity of 97%, a specificity of 91.4%, a PPV of 91.4%, and a NPV of 96.9% (Table 5). A univariate logistic regression analysis demonstrated a strong association between BPI levels > 10 ng/ml and CPPE, with a high odds ratio of 341.3 (*p* < 0.001) (Table 5). In addition, CPPE patients can be further sub-grouped into empyema patients with pus collection in the pleural space. Among the 33 patients with CPPE in our study, 21 patients had empyema. The mean BPI level of the empyema group was 332.0 ng/ml, which was significantly higher (*p* = 0.001) than in the non-purulent CPPE patients (173.7 ng/ml) (Supplementary Table 5). These observations may indicate that the BPI levels are elevated in exacerbated disease. We also evaluated the performance of these four biomarkers in identifying which patients with a PPE required drainage. Supplementary Table 6 showed that the AUCs for identifying PPE patients who required chest drainage were 0.793 for BPI, 0.756 for NGAL, 0.760 for AZU1, and 0.768 for calprotectin.

**Table 5:** Diagnostic accuracy of individual pleural fluid tests for distinguishing CPPE as compared with UPPE.

Biomarker Candidates	Cutoff	Sensitivity (%)	Specificity (%)	PPV (%)	NPV (%)	OR (95% confidence interval)	AUC (95% confidence interval)
BPI	> 10 ng/ml	97.0	91.4	91.4	97.0	341.3 (33.7, 3458.1)	0.966 (0.925–1.000)
NGAL	> 600 ng/ml	75.8	94.3	92.6	80.5	51.6 (10.1, 264.3)	0.875 (0.788–0.963)
AZU1	> 175 ng/ml	97.0	65.7	72.7	95.8	61.3 (7.4, 505.5)	0.807 (0.700–0.914)
Calprotectin	> 90 μg/ml	81.8	94.3	93.1	84.6	74.3 (13.9, 398.1)	0.937 (0.879–0.995)
LDH	> 1000 U/l	81.8	100	100	85.4	144 (48.0, 431.9)	0.915 (0.836–0.994)
Glucose	< 60 mg/dl	75.8	100	100	81.4	104 (35.1, 307.8)	0.907 (0.829–0.985)
pH	< 7.2	75.8	100	100	81.4	104 (35.1, 307.8)	0.947 (0.898–0.996)

PPV: positive predictive values; NPV: negative predictive values; AUC: area under ROC curve; BPI: bactericidal permeability-increasing protein; NGAL: neutrophil gelatinase-associated lipocalin; AZU1: azurocidin. The parapneumonic effusion (PPE, refer to UPPE and CPPE) and non-parapneumonic effusion (non-PPE, refer to transudates, other exudates, and malignant) in this study.

### The combination of pleural fluid BPI levels with LDH levels for the diagnosis of CPPE

**Figure 3:**
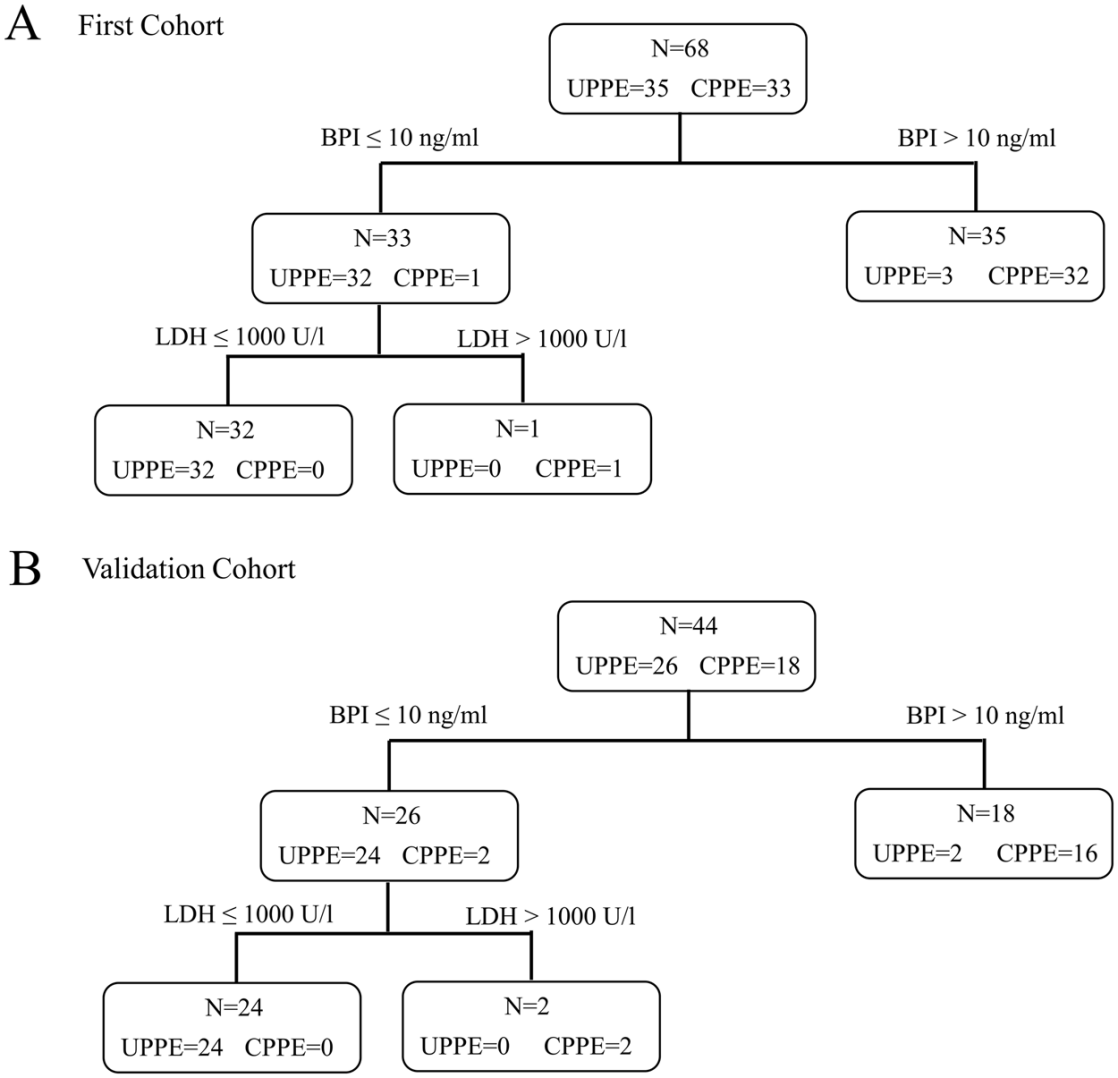
The diagnostic power of the pleural fluid BPI levels combined with LDH levels for identifying complicated parapneumonic effusions. A decision tree for CPPE and UPPE discrimination in the first cohort (**A**) and in the validation cohort (**B**). In the first cohort, the sensitivity and specificity of the combination of the pleural fluid BPI levels and LDH levels were 100% and 91.4%, respectively. In the second cohort, the sensitivity and specificity of the combination of the pleural fluid BPI levels and LDH levels were 100% and 92.3%, respectively.

Supplementary Table 7 showed the correlation between these four novel biomarkers and the biochemical parameters. All parameters studied in pleural effusions showed a statistically significant correlation (*p* < 0.01). In the case of pH and glucose, the correlation with respect to the other parameters was negative (Supplementary Table 7). Among the four novel biomarkers identified, the pleural fluid levels of BPI showed the highest positive correlation with LDH (*r* = 0.73) and a negative correlation with pH (*r* = −0.83) and glucose (*r* = −0.77) (Supplementary Table 7). Among the three biochemical parameters, the pleural fluid levels of LDH had the best diagnostic value for CPPE, with a sensitivity of 81.8%, a specificity of 100%, a PPV of 100%, and a NPV of 85.4%. Because the BPI levels and the biochemical parameter were also sensitive and specific for the diagnosis of CPPE, we sought to combine the BPI levels and LDH levels to improve the diagnostic power of BPI measurement for clinical use. The optimal cutoff BPI levels in the pleural fluid was 10 ng/ml; the test was considered positive for BPI levels > 10 ng/ml. According to this criterion, 32 and 1 cases in the CPPE group (N = 33) were positive and negative, respectively. The optimal cutoff LDH levels in the pleural fluid was 1000 U/l; the test was considered positive for LDH levels > 1000 U/l. Thus, the 1 CPPE case which was negative based on detection of BPI levels was now positive by detection LDH levels (Fig. 3a). Thus, the combination of pleural fluid BPI levels with LDH levels improved the sensitivity to 100% for identifying CPPE.

A total of 44 patients from a new independent cohort were collected and pleural fluid levels of these biomarkers were analyzed (Supplementary Table 8). As shown in Table 3, the pleural fluid levels of BPI, NGAL, AZU1, and calprotectin in CPPE were significantly higher than those in UPPE. Consistent with the results we observed in the first cohort, the AUCs for distinguishing CPPE from UPPE were 0.972 for BPI, 0.865 for NGAL, 0.848 for AZU1, 0.968 for calprotectin in the validation cohort (Supplementary Table 8). The performance of the BPI levels combined with LDH levels for identifying CPPE was consistent with the first cohort, with a sensitivity of 100% and a specificity of 92.3% in the validation cohort (Fig. 3b). In conclusion, we identified BPI, NGAL, AZU1 and calprotectin to be novel biomarkers for the diagnosis of CPPE. The combination of the BPI levels and LDH levels constitutes the highly sensitivity and specificity for CPPE diagnosis.

## Discussion

In this study, we provided a comprehensive proteome profiling of UPPE and CPPE fluid specimen samples. Using iTRAQ-based mass spectrometry analysis, 766 proteins were identified in our pleural effusion samples. Among these proteins, 45 proteins were quantified as upregulated proteins in CPPE. The four novel proteins (BPI, NGAL, AZU1, and calprotectin) identified in pleural effusion fluid specimens were further verified by ELISA in samples from 176 patients with different pleural effusion aetiologies. Patients with PPE expressed significantly higher levels of BPI, NGAL, AZU1, and calprotectin than patients with other aetiologies. In addition, our test data using the univariate logistic regression analysis demonstrated a strong link between patients with a BPI level > 10 ng/ml and CPPE. Compared with currently used biochemical parameters, such as LDH, glucose, and pH, the distinct characteristics of BPI achieved better performance in identifying CPPE, with an AUC value of 0.966. Furthermore, the combination of pleural fluid BPI levels with LDH levels improved the sensitivity to 100% for identifying CPPE.

In our effusion proteome datasets, we identified the elevated expression of certain proteins that have previously been reported in CPPE, including neutrophil collagenase (also called matrix metalloproteinase 8 (MMP8)), neutrophil elastase, matrix metalloproteinase 9 (MMP9), and MPO. Therefore, the high-throughput proteomics approach not only assisted in verifying the reported molecules but also performed as a tool to discover new biomarkers for PPEs.

LDH is composed of four subunits and a total of five distinct LDH isoenzymes with a different combination of two common subunits are expressed. In our analysis, it is indeed highly elevated in the pleural fluid of a total of 33 CPPE patients (5781.88 ± 1744.09 U/l) than that of UPPE patients (401.14 ± 52.05 U/l) by ELISA analysis for total LDH (Table 2). The samples used for the proteomic analysis were from randomly selected 4 CPPE patients and 4 UPPE patients whose average LDH concentrations were 1264.25 ± 440.08 U/l and 789.75 ± 45.37 U/l, respectively (Supplementary Table 1). The difference of LDH concentration among these two groups of patients is about 1.6 fold. The results of our proteomic analysis showed that the amounts of LDH-A chain are up-regulated 1.47 fold (Exp 1) and 1.72 fold (Exp 2) in the samples of these 4 CPPE patients (Supplementary Table 2). These data are in good agreement with the ELISA results suggesting highly compatible and reliable comparison between the proteomic analysis and ELISA analysis. The reason that LDH holds one of the lowest upregulated protein fold changes in the detected proteome is simply due to the use of selected samples.

BPI and AZU1 are neutrophil granule proteins that have antimicrobial activities against bacteria^14, 15^. Many reports indicate that BPI and AZU1 may play a role in infectious and inflammatory diseases^16, 17^. Plasma levels of BPI and AZU1 are increased in severe sepsis^18, 19^. High plasma levels of BPI correlate with increased mortality during sepsis, indicating massive bacterial counts^19^. However, the levels of BPI or AZU1 in pleural effusions have not been addressed. In our study, we demonstrated that pleural BPI had the best diagnostic value for CPPE (AUC = 0.966). We also found that the levels of BPI in pleural effusion were two-fold higher in patients with empyema; therefore, BPI may be a marker for disease progression. The longitudinal cohort study will help us to further characterize whether the elevated BPI levels indicate more severe disease. The AUC for distinguishing PPE from non-PPE was 0.943 for AZU1. The sensitivity of AZU1 for distinguishing CPPE from UPPE was 97.0%, which is similar to BPI. However, the specificity of AZU1 (65.7%) was not sufficient for clinical use.

NGAL is a neutrophil granule protein involved in the immune response. An elevated level of NGAL in serum has been reported in kidney disease, inflammatory diseases, heart diseases, metabolic diseases, and cancers^20–23^. In our study, we characterized PPE into two subtypes (UPPE and CPPE) and found that the pleural fluid levels of NGAL were significantly higher in CPPE than UPPE (*p * <0.001). A novel finding in our study was that the pleural fluid NGAL levels can not only distinguish PPE from non-PPE but also can distinguish CPPE from UPPE. Calprotectin is a neutrophil protein that plays a role in inflammatory disease and cancer^24–26^. In pneumonia patients, calprotectin levels are elevated in BALF, lung tissue and serum^27^. A novel finding in our study was that the level of pleural calprotectin was significantly elevated in PPE, particularly CPPE. Additionally, the mean calprotectin level of the empyema group was 168.6 ng/ml, which was slightly higher (*p* = 0.013) than that of non-purulent CPPE patients (124.8 ng/ml) (Supplementary Table 5). These observations may indicate that calprotectin levels are slightly elevated in exacerbated disease.

The strong correlations between these four neutrophil proteins and the glucose, LDH, and pH levels of pleural fluid indicate that specific proteins may play a role in the modulation of infection in the pleural fluid of PPEs. These findings provide important insights into the role of neutrophils in CPPE pathogenesis. During the acute phase of infectious pleural effusions, neutrophils are the first leukocytes to be recruited to the inflamed tissues, leading to elevated neutrophil counts in pleural effusions^28^. In our study, the percentage of neutrophils in total nucleated cells was significantly higher in CPPEs (84.8 ± 2.7%) than in UPPEs (68.1 ± 3.1%) (Data not shown). However, using the neutrophil count to distinguish UPPEs from CPPEs is less effective statistically than using the four proteins (BPI, NGAL, AZU1, and calprotectin) we identified as biomarkers in clinical diagnosis. Because the half-life of the neutrophil is short but the proteins released from the dead neutrophils may still continue to activate the inflammatory response, evaluating only the neutrophil count in pleural effusions will not accurately identify the severity of the inflammation status during the infection. Thus, measuring the levels of proteins released by neutrophils in pleural effusions will lead to a more accurate clinical diagnosis than measuring the neutrophil count when diagnosing PPE. To our knowledge, this is the first clinical quantitative proteomic study to profile the proteome of UPPEs and CPPEs using iTRAQ-based mass spectrometry technology. This high-throughput proteomics approach provides an opportunity to discover new biomarkers that have not been previously reported for UPPE and CPPE.

The study presented herein has some limitations. The first limitation is the lack of pulmonary tuberculosis patients enrolled in this study. Second, the treatment for CPPE and loculated PPE may require aggressive pleural drainage, but the decision to perform the drainage treatment primarily depended on the patients and the families in the present study, which influenced the eventual outcomes.

In conclusion, the present study reveals that pleural levels of BPI, NGAL, AZU1, and calprotectin are significantly associated with PPEs, particularly CPPEs, according to high-throughput proteomics analysis. Our present findings not only provide a panel of novel biomarkers for a better identification of CPPE but also illustrate the opportunity of this effective tool for shedding lights on the potential immune-pathological mechanisms involved in this high-risk infectious disease.

## Methods

### Study subjects and study design

In total, 220 pleural effusion samples of diagnosed patients from two teaching hospitals were included in this prospective study, which was approved by the Institutional Review Board at the Chang Gung Memorial Hospital (CGMH) and Tri-Service General Hospital (TSGH), Taiwan. All experiments were performed in accordance with the guidelines and regulations by the Institutional Review Board at CGMH and TSGH. Prior to sample collection, written informed consent was obtained from all patients and/or their families. The pleural effusion samples were collected and diagnosed when the patients were admitted to the hospital, followed by ultrasound-guided thoracentesis. Patients were classified in the first cohort into five groups (Table 2) according to the cause of pleural effusion: UPPE (35 patients), CPPE (33 patients), transudates (37 patients), other exudates (32 patients), and malignant effusions (39 patients). A total of 44 patients from a new independent cohort were collected: UPPE (26 patients) and CPPE (18 patients). Based on the LIGHT criteria, effusions were categorized as either exudates or transudates^11^. PPE is defined as any exudative effusion associated with pneumonia, lung abscess, or bronchiectasis and is further classified as UPPE or CPPE. CPPE is defined as the presence of PPE with one of the following additional criteria: (1) pH < 7.2; (2) glucose < 60 mg/dl; (3) LDH > 1000 U/l; (4) bacteria found on Gram’s stain or culture; or (5) frank pus^3, 12^. Patients received pleural drainage or surgery for the treatment of their PPEs based on previously accepted guidelines [3]. Malignant pleural effusions were diagnosed based on the presence of positive malignant cells in a cytological examination. After collection, effusions were centrifuged at 3,000 rcf for 10 min. Acellular supernatants were collected and stored at −80 °C until used for further experiments. For the initial discovery phase, a set of effusions from UPPE (4 patients) and CPPE (4 patients) patients were screened using isobaric tags for relative and absolute quantitation (iTRAQ)-based mass spectrometry. A total of 220 pleural effusions from the five types of PPEs were used to validate potential biomarkers by enzyme-linked immunosorbent assay (ELISA).

### Removal of high-abundance proteins of pleural effusion samples

Pleural effusion samples were depleted of six high-abundance human plasma proteins (albumin, immunoglobulin G, immunoglobulin A, transferrin, α1-antitrypsin, and haptoglobin) using a Multiple Affinity Removal System (MARS) affinity column (Hu-6HC, 4.6 × 100 mm; Agilent Technologies, Wilmington, DE, USA) on an ÄKTA Purifier-10 fast performance liquid chromatography system (FPLC; GE Healthcare/Amersham Bioscience, UK). Depleted pleural effusion samples were concentrated using an Amicon Ultra-4 centrifugal filter unit with an Ultracel-3 membrane (Millipore, Carrigtwohill, Co. Cork, Ireland). After the depletion of high-abundance proteins, the protein concentrations of the PPE samples were determined using the BCA protein assay kit from Pierce (Rockford, IL, USA). In the experiment 1, pleural effusion samples (10 μg proteins) from each 4 UPPE patients and 4 CPPE patients with high-abundance protein depletion were then pooled into UPPE group (total 40 μg proteins in UPPE) and CPPE group (total 40 μg proteins in CPPE), respectively. For the technical replicates, the second set (experiment 2) of pooled samples from 4 UPPE patients and 4 CPPE patients were prepared from different batches.

### In-solution digestion of protein and iTRAQ labeling

For tryptic digestion of PPE proteins, 30 μg proteins from the pooled UPPE group or CPPE group were reduced with 5 mM tris-(2-carboxyethyl)-phosphine (TCEP, Sigma-Aldrich, St. Louis, MO, USA) at 60 °C for 30 min, treated with 10 mM methyl methanethiosulfonate (MMTS, Sigma-Aldrich) at 25 °C for 30 min, and then digested at 37 °C for 16 hours by trypsin (Promega, Madison, WI, USA) in solution containing 100 mM triethylammonium bicarbonate (TEABC, Sigma-Aldrich). For iTRAQ labeling, the tryptic peptides were labelled with four different iTRAQ labelling reagents according to the manufacturer’s protocols. Briefly, the peptides from the pooled UPPE group and pooled CPPE group (Exp 1) were labeled with iTRAQ 114 and 115 tags, respectively. For the technical replicates, the iTRAQ 116 and 117 tags were respectively incubated with peptide samples from the pooled UPPE group and pooled CPPE group (Exp 2), respectively, which were prepared in different batches. After incubation for 1 h at room temperature, the four labeled samples were pooled, desalted, and then dried using vacuum centrifugation.

### Peptide fractionation and LC-MS/MS analysis

The iTRAQ-labeled peptide mixtures (30 μg) were separated using the on-line 2D-HPLC system (Dionex Ultimate 3000, Thermo Fisher Scientific, San Jose, CA, USA) and analyzed using a method described previously^29^. Briefly, the desalted peptides (30 μg) were reconstituted in 50 μL of buffer A (0.1% formic acid and 30% acetonitrile) and loaded onto the homemade column (Luna SCX, 5 μm, 0.5 × 180 mm) at a flow rate of 5 μL/min for 30 min. The peptides were then eluted with a 0–100% gradient of buffer B (0.5 M ammonium chloride, 30% acetonitrile, and 0.1% formic acid). The resulting 66 peptide fractions were diluted in-line prior to trap onto the column Zorbax 300SB-C18 (0.3 × 5 mm, Agilent Technologies, Wilmington, DE, USA). Each fraction was then separated on a homemade column (HydroRP 2.5 μm, 75 μm inner diameter and 20 cm length) with a 15-μm tip using buffer C (acetonitrile containing 0.1% formic acid). A linear gradients of buffer C (3–28% for 37 min, 28–50% for 12 min, 50–95% for 2 min, 95% for 5 min, and 3% for 9 min) was applied at a flow rate of 0.3 μL/min.

The LC equipment was connected to the LTQ-Orbitrap ELITE Hybrid MS (Thermo Fisher Scientific) operated using Xcalibur software (Version 2.2, Thermo Fisher Scientific). Full-scan MS was performed in the Orbitrap MS over a range of 400 to 2000 Da and a resolution of 60000 at *m/z* 400. The ion signal of (Si(CH_3_)_2_O)_6_H^+^ at *m/z* 445.120025, 462.146574, and 536.165365 was used for lock masses and internal calibration. The 12 data-dependent MS/MS scan events, including 6 collision-induced dissociations (CID) acquired in LTQ MS and 6 higher-energy collision-induced dissociations (HCD) acquired in Orbitrap MS, were followed by one MS scan for the six most abundant ions in the preview MS scan. The *m/z* values selected for MS2 were excluded dynamically for 40 seconds with a relative mass window of 1.5 Da. The electrospray voltage was set to 1.8 kV, and the temperature of the capillary was set to 220 °C. Automatic gain control was applied to preclude over-filling of the ion trap, and 2 × 10^6^ ions/1000 ms, 5 × 10^3^ ions/150 ms, and 3 × 10^4^ ions/300 ms were set as the maximum accumulated ions/time for the full scan, CID, and HCD, respectively.

### Protein database search and quantitative data analysis

The data analysis was carried out using Proteome Discoverer software (version 1.4, Thermo Fisher Scientific, San Jose, CA, USA) including the reporter ions quantifier node for iTRAQ quantification. The MS/MS spectra was searched against the Swiss-Prot human sequence database (released on Apr 16, 2014, selected for Homo sapiens, 20265 entries) using the Mascot search engine (Matrix Science, London, UK; version 2.2.04). For protein identification, 10 ppm mass tolerance was permitted for intact peptide masses and 0.5 Da for fragment ions, with allowance for one missed cleavages made from the trypsin digest: oxidized methionine (+16 Da) as a potential variable modification, and iTRAQ (N terminal, +144 Da), iTRAQ (K, +144 Da), and methyl methanethiosulfonate (C, +46 Da) as the fixed modifications. Data were then filtered based on high confidence of peptide identification to ensure an overall false discovery rate below 0.01. The identification of epithelial keratins was excluded. Proteins with single peptide hits were removed, and quantitative data were exported from Proteome Discoverer and manually normalized such that the log2 of iTRAQ ratios displayed a median value of zero for all peptides in a given protein. This was performed across an entire labeling experiment to correct for variation in protein abundance.

The cutoff value for determining whether a protein in considered dysregulated was selected according to the analysis using comparison of protein levels between the same UPPE samples in two different batch experiments; the range of protein ratios (iTRAQ 116/114) from 0.715 (mean minus 3 SD) to 1.351 (mean plus 3 SD) covers 99% of the proteins. Proteins quantified with ratios between 0.715–1.351 were considered as no change in our analysis. Therefore, proteins whose CPPE/UPPE ratios were higher than the mean plus one SD (1.381 and 1.593 in Exp 1 and Exp 2, respectively) or lower than the mean minus one SD (0.658 and 0.668 in Exp 1 and Exp 2, respectively) were considered potential dysregulated proteins.

### Bioinformatics analysis

Biological process classification and signalling pathway analysis for the dysregulated proteins were performed with the tools of the Database for Annotation, Visualization and Integrated Discovery (DAVID, version 6.7, http://david.abcc.ncifcrf.gov/) and the Kyoto Encyclopedia of Genes and Genomes (KEGG) database (http://www.genome.jp/kegg/pathway.html). After relative quantification analysis, differentially expressed proteins were uploaded into MetaCore version 6.13 build 61585 (GeneGo, St. Joseph, MI) for gene ontology (GO) of cellular processes analysis.

### Measurement of proteins by ELISA

Commercial sandwich ELISA kits were used to detect the pleural effusion levels of bactericidal permeability-increasing protein (BPI; LSBio, WA, USA), neutrophil gelatinase-associated lipocalin (NGAL; R&D Systems, MN, USA), azurocidin (AZU1; Abnova, CA, USA), and calprotectin (R&D Systems, MN, USA). The assays were performed according to the manufacturer’s guidelines.

### Statistical analyses

Between-group comparisons were performed with a nonparametric Mann-Whitney U test for two groups. Receiver operator characteristic (ROC) curves were generated to illustrate the decision value of various cut-off points for BPI, NGAL, AZU1, and calprotectin. The point with the largest sum of specificity and sensitivity was selected as the threshold. The positive predictive values (PPVs) and negative predictive values (NPVs) were calculated. Spearman correlation was used to measure the association between biochemical parameters and candidate proteins. A univariate logistic regression was performed to analyse the association between pleural fluid tests and the presence of CPPE. Unadjusted ORs were calculated as an estimate of risk. All data were processed using SPSS software version 12.0 (SPSS Inc., Chicago, IL, USA). A *p* value < 0.05 was considered statistically significant.

### Data availability

The effusion proteome datasets in this study are available from the corresponding author (C.Y.Y.) on reasonable request.

## Electronic supplementary material

Proteome profiling reveals novel biomarkers to identify complicated parapneumonic effusions

## References

[1] Light RW (2006). Parapneumonic effusions and empyema. Proc Am Thorac Soc.

[2] Sahn SA (2007). Diagnosis and management of parapneumonic effusions and empyema. Clin Infect Dis.

[3] Davies HE, Davies RJ, Davies CW (2010). & British Thoracic Society Pleural Disease Guideline Group Management of pleural infection in adults: British Thoracic Society Pleural Disease Guideline 2010. Thorax.

[4] Alegre J (2002). Pleural-fluid myeloperoxidase in complicated and noncomplicated parapneumonic pleural effusions. Eur Respir J.

[5] Aleman C (2003). Polymorphonuclear elastase in the early diagnosis of complicated pyogenic pleural effusions. Respiration.

[6] Porcel JM, Vives M, Esquerda A (2004). Tumor necrosis factor-alpha in pleural fluid: a marker of complicated parapneumonic effusions. Chest.

[7] Iglesias D (2005). Metalloproteinases and tissue inhibitors of metalloproteinases in exudative pleural effusions. Eur Respir J.

[8] Porcel JM (2009). Biomarkers of infection for the differential diagnosis of pleural effusions. Eur Respir J.

[9] Marchi E (2012). Proinflammatory and antiinflammatory cytokine levels in complicated and noncomplicated parapneumonic pleural effusions. Chest.

[10] Porcel JM (2010). Pleural fluid tests to identify complicated parapneumonic effusions. Curr Opin Pulm Med.

[11] Yu CJ (2011). Comprehensive proteome analysis of malignant pleural effusion for lung cancer biomarker discovery by using multidimensional protein identification technology. J Proteome Res.

[12] Wu CC, Chu HW, Hsu CW, Chang KP, Liu HP (2015). Saliva proteome profiling reveals potential salivary biomarkers for detection of oral cavity squamous cell carcinoma. Proteomics.

[13] Liu PJ (2015). In-depth proteomic analysis of six types of exudative pleural effusions for nonsmall cell lung cancer biomarker discovery. Mol Cell Proteomics.

[14] Levy O (2000). A neutrophil-derived anti-infective molecule: bactericidal/permeability-increasing protein. Antimicrob Agents Chemother.

[15] Linder A, Soehnlein O, Akesson P (2010). Roles of heparin-binding protein in bacterial infections. J Innate Immun.

[16] Schultz H, Weiss JP (2007). The bactericidal/permeability-increasing protein (BPI) in infection and inflammatory disease. Clin Chim Acta.

[17] Soehnlein O, Lindbom L (2009). Neutrophil-derived azurocidin alarms the immune system. J Leukoc Biol.

[18] Linder A (2012). Elevated plasma levels of heparin-binding protein in intensive care unit patients with severe sepsis and septic shock. Crit Care.

[19] Rintala E, Peuravuori H, Pulkki K, Voipio-Pulkki LM, Nevalainen T (2000). Bactericidal/permeability-increasing protein (BPI) in sepsis correlates with the severity of sepsis and the outcome. Intensive Care Med.

[20] Otto GP (2015). Plasma Neutrophil Gelatinase-Associated Lipocalin Is Primarily Related to Inflammation during Sepsis: A Translational Approach. PLoS One.

[21] de Geus HR, Bakker J, Lesaffre EM, le Noble JL (2011). Neutrophil gelatinase-associated lipocalin at ICU admission predicts for acute kidney injury in adult patients. Am J Respir Crit Care Med.

[22] Oikonomou KA (2012). Neutrophil gelatinase-associated lipocalin (NGAL) in inflammatory bowel disease: association with pathophysiology of inflammation, established markers, and disease activity. J Gastroenterol.

[23] Haase M (2009). Accuracy of neutrophil gelatinase-associated lipocalin (NGAL) in diagnosis and prognosis in acute kidney injury: a systematic review and meta-analysis. Am J Kidney Dis.

[24] Khammanivong A (2016). Involvement of calprotectin (S100A8/A9) in molecular pathways associated with HNSCC. Oncotarget.

[25] Sanchez-Otero N (2012). Calprotectin: a novel biomarker for the diagnosis of pleural effusion. Br J Cancer.

[26] Gebhardt C, Nemeth J, Angel P, Hess J (2006). S100A8 and S100A9 in inflammation and cancer. Biochem Pharmacol.

[27] Achouiti A (2014). Myeloid-related protein-8/14 facilitates bacterial growth during pneumococcal pneumonia. Thorax.

[28] Corcoran JP (2015). Pleural infection: past, present, and future directions. Lancet Respir Med.

[29] Chen CD (2014). Targeted proteomics pipeline reveals potential biomarkers for the diagnosis of metastatic lung cancer in pleural effusion. J Proteome Res.

