# Supplementary material for: Proteome profiling reveals novel biomarkers to identify complicated parapneumonic effusions
Source: Sci Rep. 2017 Jun 22;7:4026. doi: 10.1038/s41598-017-04189-4 (PMC5481374; doi:10.1038/s41598-017-04189-4)
Supplement: Supplementary file 1 — Proteome profiling reveals novel biomarkers to identify complicated parapneumonic effusions [file 41598_2017_4189_MOESM1_ESM.pdf]

Supplementary Information

Proteome profiling reveals novel biomarkers to identify complicated parapneumonic effusions

Kuo-An Wu, Chih-Ching Wu, Chi-De Chen, Chi-Ming Chu, Li-Jane Shih, Yu-Ching Liu, Chih-Liang Wang, Hsi-Hsien Lin, and Chia-Yu Yang

Supplementary Figure

A

Proteins	Exp 1	Exp 2
Identification	766	766
Quantification	740	738

B

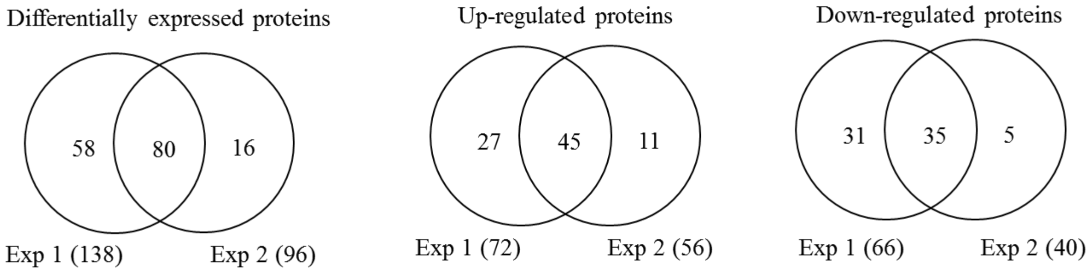

Supplementary Fig 1. Protein identification and quantification in UPPE and CPPE using iTRAQ-based analysis.

(A) The protein identification matched ProteinProphet probability higher than 0.95 and with at least two unique peptides was used. The protein quantification was performed using Libra module in the TTP platform. (B) Relatively protein quantified was calculated as the ratio of CPPE to UPPE. The ratio > mean + 1SD was considered as up regulation in CPPE, whereas the ratio < mean - 1SD was considered as down regulation in CPPE. Venn diagrams showed overlaps between the effusion proteins identified in the two experiment. The differentially expressed proteins, up-regulated proteins, and down-regulated proteins were shown in venn diagrams. The numbers of identified proteins are listed.

Supplementary Tables

Supplementary Table 1. Clinical characteristics of 8 patients used to establish pleural effusion proteomes using iTRAQ-based mass spectrometry

	UPPE	CPPE
Patients	4	4
male (%)	4 (100%)	4 (100%)
Age (years)[a]	51.25 ± 2.68	52.5 ± 3.09
Proteins (g/dl)[a]	4.90 ± 0.34	5.31 ± 0.36
Glucose (mg/dl)[a]	96.77 ± 22.14	39.25 ± 19.88
LDH (U/L)[a]	789.75 ± 45.37	1264.25 ± 440.08
pH[a]	7.32 ± 0.03	7.17 ± 0.09

[a] Data are presented as mean ± s.e.m.

UPPE: uncomplicated parapneumonic effusion; CPPE: complicated parapneumonic effusion

Supplementary Table 2. List of 45 up-regulated proteins in CPPE compared with UPPE

Gene Name	Accession No.[a]	Protein Name	Up-regulated[b]	Exp 1 (Fold)[c]	Exp 2 (Fold)[d]	Number of unique peptides	Number of total peptides	Number of total spectra	Sequence coverage (%)	Mascot score	MW (KDa)	Peptide sequence	Modification	Calculated MH+	IonScore (HCD)	IonScore (CID)
LPA	P08519	Apolipoprotein	Up	3.56	3.48	17	17	49	29.09	933	501.0	SPVVQDCYHGDGR	N-Term(iTRAQ); C7(Methy)	1622.71	83	48.15
												EAQLLVIENEVCNHYK	N-Term(iTRAQ); C12(Methy); K16(iTRAQ)	2236.13	69.85	53.71
												ATTVTGTPCQEWAAQEPHR	N-Term(iTRAQ); C9(Methy)	2273.06	65.07	45.21
												GSFSTTVTGR	N-Term(iTRAQ)	1156.61	65.04	47.42
												LFLEPTQADIALLK	N-Term(iTRAQ); K14(iTRAQ)	1860.11	55.01	56.83
												GTFSTTVTGR	N-Term(iTRAQ)	1170.62	52.47	52.03
												FVTWIEGMMR	N-Term(iTRAQ)	1413.72	52.32	48.98
												TPEYYPNAGLIMNYCR	N-Term(iTRAQ); C15(Methy)	2094.94	49.44	36.8
												YICAEHLAR	N-Term(iTRAQ); C3(Methy)	1265.62	45.26	28.66
												GTLSTTITGR	N-Term(iTRAQ)	1150.65	44.57	49.71
												NPDAVAAPYCYTR	N-Term(iTRAQ); C10(Methy)	1630.75	43.37	63.72
												GTYSTTVTGR	N-Term(iTRAQ)	1186.62	39.04	63.85
												RIPLYYPNAGLTR	N-Term(iTRAQ)	1677.96	28.47	21.42
												HSTFIPGTNK	N-Term(iTRAQ); K10(iTRAQ)	1389.77	29.93	29.46
												TPENYPNAGLTR	N-Term(iTRAQ)	1476.75	29.85	-
												LSRPAVITDK	N-Term(iTRAQ); K10(iTRAQ)	1387.85	19.79	16.99
												TPEYYPNAGLIMNYCR	N-Term(iTRAQ); M12(Oxida); C15(Methy)	2110.95	19.39	14.29
												WAGLEK	N-Term(iTRAQ); K6(iTRAQ)	991.58	31.62	34.83
REG1A	P05451	Lithostathine-1-alpha	Up	3.30	3.07	3	3	11	30.12	249	18.7	SWGIGAPSSVNPGYCVSLTSSTGFQK	N-Term(iTRAQ); C15(Methy); K26(iTRAQ)	2964.45	73.9	60.36
												ESGTDDFNVWIGLHDPK	N-Term(iTRAQ); K17(iTRAQ)	2218.11	55.52	34.91
												DVPCEDK	N-Term(iTRAQ); C4(Methy);	1139.53	38.59	36.16
HIST1H2BJ	P06899	Histone H2B type 1-J	Up	2.81	2.16	2	7	50	46.03	1389	13.9	AMGIMNSFVNDIFER	N-Term(iTRAQ)	1887.91	104.27	81.8
												AMGIMNSFVNDIFER	N-Term(iTRAQ); M5(Oxida)	1903.91	93.02	66.33
												QVHPDTGISSK	N-Term(iTRAQ); K11(iTRAQ)	1456.80	67.45	63.26
												LLLPGELAK	N-Term(iTRAQ); K9(iTRAQ)	1241.81	62.05	48.14
												AMGIMNSFVNDIFER	N-Term(iTRAQ); M2(Oxida); M5(Oxida)	1919.91	53.84	23.17
												ESYSIYVYK	N-Term(iTRAQ); K9(iTRAQ)	1439.77	48.94	38.35
												EIQTAVR	N-Term(iTRAQ)	960.56	41.85	64.56
												KESYSIYVYK	N-Term(iTRAQ); K1(iTRAQ); K10(iTRAQ)	1711.96	27.91	43.18
												LLLPGELAK	K9(iTRAQ)	1097.71	44.36	-
												LAHYNK	N-Term(iTRAQ); K6(iTRAQ)	1033.60	38.34	37.42
HMGB2	P26583	High mobility group protein B2	Up	2.75	3.00	3	3	5	14.35	135	24.0	LGEMWSEQSAK	N-Term(iTRAQ); M4(Oxida); K11(iTRAQ)	1569.78	47.31	50.89
												SEHPGLSIGDTAK	N-Term(iTRAQ); K13(iTRAQ)	1599.86	46.84	36.8
												NYVPPK	N-Term(iTRAQ); K6(iTRAQ)	1005.60	-	37.8
BPI	P17213	Bactericidal permeability-increasing protein	Up	2.68	3.43	6	6	14	17.25	381	53.9	GLDYASQQGTAALQK	N-Term(iTRAQ); K15(iTRAQ)	1838.99	73.63	79.66
												IPDYSDSFK	N-Term(iTRAQ); K9(iTRAQ)	1359.70	67.61	25.09
												VQLYNVVLQPHQNFLLFGADVVYK	N-Term(iTRAQ); K24(iTRAQ)	3092.72	45.3	21.32
												GFPLPTPAR	N-Term(iTRAQ)	1099.64	44.91	36.41
												EFQLPSSQISMVPNVGLK	N-Term(iTRAQ); K18(iTRAQ)	2262.24	35.75	-
												FSISNANIK	N-Term(iTRAQ); K9(iTRAQ)	1281.74	-	55.33
MMP8	P22894	Neutrophil collagenase	Up	2.67	2.86	18	18	74	47.97	1568	53.4	ISQGEADINIAFYQR	N-Term(iTRAQ)	1868.96	95.31	69.86
												VDAVFQQEHFFHVFSGPR	N-Term(iTRAQ)	2291.15	80.2	72.07
												SISGAFPGIESK	N-Term(iTRAQ); K12(iTRAQ)	1480.83	75.23	82.96
												QFMEPGYPK	N-Term(iTRAQ); K9(iTRAQ)	1384.72	64.75	50.1
												DISNYGFPSSVQAIDAAVFYR	N-Term(iTRAQ)	2464.23	59.89	29.81
												GNQYWALSGYDILQGYPK	N-Term(iTRAQ); K18(iTRAQ)	2361.21	58.11	19.52
												AIKDAFELWSVASPLIFTR	N-Term(iTRAQ); K3(iTRAQ)	2452.39	57.65	38.89
												DAFELWSVASPLIFTR	N-Term(iTRAQ)	1996.07	54.22	46.95

												TVQDYLEK	N-Term(iTRAQ); K8(iTRAQ)	1283.71	52.84	60.04
												CGVPDSGGFMLTPGNPK	C1(Methy); N-Term(iTRAQ); M10(Oxida); K17(iTRAQ)	2026.96	48.68	44.54
												CGVPDSGGFMLTPGNPK	N-Term(iTRAQ); C1(Methy);	2010.97	48.34	41.39
												NYTPQLSEAEVER	N-Term(iTRAQ)	1679.84	44.28	62.76
												FYQLPSNQYQSTR	N-Term(iTRAQ)	1775.88	43.28	39.89
												GEILFFK	N-Term(iTRAQ); K7(iTRAQ)	1141.69	42.37	43.23
												QFMEPGYPK	N-Term(iTRAQ); M3(Oxida);	1400.71	42.34	34.42
												SISGAFPGIESKVDAVFQQEHFFHVFSGPR	N-Term(iTRAQ); K12(iTRAQ)	3608.85	39.46	18.98
												TYFFVNDQFWR	N-Term(iTRAQ)	1666.81	38.71	36.83
												YYAFDLIAQR	N-Term(iTRAQ)	1403.74	30.25	-
												VEMNFISLFWPSLPTGIQAAYEDFDRDLIFLFK	N-Term(iTRAQ); K33(iTRAQ)	4211.18	29.31	-
												DLIFLFK	N-Term(iTRAQ); K7(iTRAQ)	1183.72	42.62	37.34
ELANE	P08246	Neutrophil elastase	Up	2.54	2.74	4	4	28	19.85	403	28.5	GGCASGLYPDAFAPVAQFVNWIDSIIQR	N-Term(iTRAQ); C3(Methy)	3185.57	69.75	10.05
												VVLGAHNLSR	N-Term(iTRAQ)	1209.72	53.32	43.78
												SNVCTLVR	C4(Methy)	937.46	38.75	64.03
												SNVCTLVR	N-Term(iTRAQ); C4(Methy)	1081.56	34.51	39.49
												QVFAVQR	N-Term(iTRAQ)	991.58	-	25.77
FTL	P02792	Ferritin light chain	Up	2.52	2.29	5	5	8	32.57	179	20.0	LGGPEAGLGEYLFER	N-Term(iTRAQ)	1751.91	54.5	63.61
												ALFQDIK	N-Term(iTRAQ); K7(iTRAQ)	1122.67	48.73	34.79
												KLNQALLDLHALGSAR	N-Term(iTRAQ); K1(iTRAQ)	2008.19	39.7	-
												LNQALLDLHALGSAR	N-Term(iTRAQ)	1735.99	37.5	36.94
												TDPHLCDFLETHFLDEEVK	N-Term(iTRAQ); C6(Methy);	2622.24	23.81	-
ANXA3	P12429	Annexin A3	Up	2.41	2.64	9	9	17	34.06	387	36.4	SLGDDISSETSGDFR	N-Term(iTRAQ)	1729.80	89.97	59.24
												DISQAYYTVYK	N-Term(iTRAQ); K11(iTRAQ)	1638.86	60.97	54.68
												GIGTDEFTLNR	N-Term(iTRAQ)	1366.70	56.34	40.48
												DIVDSIK	N-Term(iTRAQ); K7(iTRAQ)	1077.64	46.41	45.14
												NTPAFLAER	N-Term(iTRAQ)	1162.63	40.41	35.67
												GDLSGHFEHLMVALVTPPAVFDAK	N-Term(iTRAQ); M11(Oxida); K24(iTRAQ)	2855.51	33.98	46.67
												SEIDLLDIR	N-Term(iTRAQ)	1217.68	33.75	31.8
												WGTDEDKFTEILCLR	N-Term(iTRAQ); K7(iTRAQ);	2160.07	14.09	12.43
												MLISILTER	N-Term(iTRAQ)	1219.71	24.68	-
MB	P02144	Myoglobin	Up	2.39	2.08	3	3	5	29.22	130	17.2	HGATVLTALGGILK	N-Term(iTRAQ); K14(iTRAQ)	1639.01	51.91	41.92
												HPGDFGADAQGAMNK	N-Term(iTRAQ); M13(Oxida); K15(iTRAQ)	1819.86	47.61	37.99
												YLEFISECIIQVLQSK	N-Term(iTRAQ); C8(Methy);	2247.20	-	28.3
FTH1	P02794	Ferritin heavy chain	Up	2.35	7.51	3	3	9	16.39	233	21.2	IFLQDIK	N-Term(iTRAQ); K7(iTRAQ)	1164.72	49.87	38.92
												QNYHQDSEAAINR	N-Term(iTRAQ)	1689.81	49.77	61.58
												ELGDHVTNLR	N-Term(iTRAQ)	1297.70	45.57	41.16
PLBD1	Q6P4A8	Phospholipase B-like 1	Up	2.33	2.64	2	2	4	2.53	71	63.2	WADIFSK	N-Term(iTRAQ); K7(iTRAQ)	1154.64	49.78	33.29
												VQDFMEK	N-Term(iTRAQ); M5(Oxida);	1200.62	28.44	27.68
MMP9	P14780	Matrix metalloproteinase-9	Up	2.25	2.10	23	23	88	47.52	1946	78.4	LWCATTSNFDSDK	N-Term(iTRAQ); C3(Methy);	1821.83	104.48	67.62
												SLGPALLLLQK	N-Term(iTRAQ); K11(iTRAQ)	1440.94	92.01	92.54
												QLSLPETGELDSATLK	N-Term(iTRAQ); K16(iTRAQ)	1990.10	81.1	68.04
												DADIVIQFGVAEHGDGYPFDGK	N-Term(iTRAQ); K22(iTRAQ)	2638.30	80.7	41.09
												LGLGADVAQVTGALR	N-Term(iTRAQ)	1584.91	77.77	70.4
												ADSTVMGGNSAGELCVFPFTFLGK	N-Term(iTRAQ); C15(Methy); K24(iTRAQ)	2782.35	74.82	49.91
												FGNADGAACHFPFIFEGR	N-Term(iTRAQ); C9(Methy)	2145.98	60.42	52.36
												SYSACTTDGR	N-Term(iTRAQ); C5(Methy)	1250.52	59.99	35.9
												MFPGVPLDTHDVFQYR	N-Term(iTRAQ); M1(Oxida)	2082.03	58.45	44.17
												MFPGVPLDTHDVFQYR	N-Term(iTRAQ)	2066.03	58.31	31.67
												FTEGPPLHK	N-Term(iTRAQ); K9(iTRAQ)	1313.74	51.14	41.92
												QVWVYTGASVLGPR	N-Term(iTRAQ)	1676.92	49.54	47.05

												SELNQVDQVGYVTYDILQCPED	N-Term(iTRAQ); C19(Methy)	2718.24	47.44	-
												QSTLVLFPGDLR	N-Term(iTRAQ)	1489.85	46.18	44.24
												QLAEEYLYR	N-Term(iTRAQ)	1328.69	40.71	51.01
												AFALWSAVTPLTFTR	N-Term(iTRAQ)	1825.02	35.33	32.88
												GSRPQGPFLIADK	N-Term(iTRAQ); K13(iTRAQ)	1673.96	27.87	13.99
												KLFFFSGR	N-Term(iTRAQ); K1(iTRAQ)	1289.76	26.39	42.1
												FQTFEGDLK	N-Term(iTRAQ); K9(iTRAQ)	1372.73	25.73	44.26
												WGFCPDQGYSLFLVAAHEFGHA LGLDHSSVPEALMYPMYR	N-Term(iTRAQ); C4(Methy); M35(Oxida); M38(Oxida)	4734.21	14.44	-
												CGVPDLGR	N-Term(iTRAQ); C1(Methy)	1006.49	31.83	-
												FGFCPSER	N-Term(iTRAQ); C4(Methy)	1132.50	25.53	-
												AYFCQDR	N-Term(iTRAQ); C4(Methy)	1092.47	-	25.81
												GVVVPTR	N-Term(iTRAQ)	871.55	30.57	37.39
ANXA1	P04083	Annexin A1	Up	2.09	2.66	12	12	39	35.26	1093	38.7	ALTGHLEEVVLALLK	N-Term(iTRAQ); K15(iTRAQ)	1894.16	97.28	88.28
												TPAQFDADELR	N-Term(iTRAQ)	1406.70	60.16	23.64
												GVDEATIIDILTK	N-Term(iTRAQ); K13(iTRAQ)	1675.97	56.83	66.95
												NALLSLAK	N-Term(iTRAQ); K8(iTRAQ)	1117.72	56.41	45.09
												KGTDVNVFNTILTTR	N-Term(iTRAQ); K1(iTRAQ)	1967.11	54.8	65.2
												GTDVNVFNTILTTR	N-Term(iTRAQ)	1694.92	53.11	50.02
												CLTAIVK	N-Term(iTRAQ); C1(Methy);	1081.63	50.78	43.31
												DITSDTSGDFR	N-Term(iTRAQ)	1357.64	49.12	38.03
												GLGTDEDTLIEILASR	N-Term(iTRAQ)	1846.98	48.92	72.88
												SEIDMNDIK	N-Term(iTRAQ); K9(iTRAQ)	1352.69	43.82	30.18
												ALYEAGER	N-Term(iTRAQ)	1052.55	31.65	37.68
												ILVALCGGN	N-Term(iTRAQ); C6(Methy)	1049.56	-	29.43
LCN2	P80188	Neutrophil gelatinase-associated lipocalin	Up	2.08	2.76	14	14	73	62.63	1411	22.6	TFVPGCQPGEFTLGNIK	N-Term(iTRAQ); C6(Methy);	2142.10	77.28	52.17
												VVSTNYNQHAMVFFK	N-Term(iTRAQ); M11(Oxida); K15(iTRAQ)	2089.08	74.49	32.34
												VPLQQNFQDNQFQGK	N-Term(iTRAQ); K15(iTRAQ)	2079.09	73.29	57.65
												MYATIYELKEDK	N-Term(iTRAQ); M1(Oxida); K9(iTRAQ); K12(iTRAQ)	1952.04	71.28	35.64
												MYATIYELK	N-Term(iTRAQ); K9(iTRAQ)	1419.78	65	63.95
												WYVVGLAGNAILR	N-Term(iTRAQ)	1575.91	63.64	45.76
												MYATIYELK	N-Term(iTRAQ); M1(Oxida);	1435.78	56.13	42.44
												TKELTSELK	N-Term(iTRAQ); K2(iTRAQ);	1480.89	54.08	32.57
												ELTSELKENFIR	N-Term(iTRAQ); K7(iTRAQ)	1766.99	44.34	25.25
												SYPGLTSYLVR	N-Term(iTRAQ)	1399.77	41.1	41.86
												TFVPGCQPGEFTLGNIK	C6(Methy); K17(iTRAQ)	1997.99	36.1	22.52
												VPLQQNFQDNQFQGKWYVVGL AGNAILR	N-Term(iTRAQ); K15(iTRAQ)	3491.88	30.66	-
												SLGLPENHIVFPVPIDQCIDG	N-Term(iTRAQ); C18(Methy)	2453.23	22.73	-
												TFVPGCQPGEFTLGNIKSYPGLT SYLVR	N-Term(iTRAQ); C6(Methy); K17(iTRAQ)	3378.75	15.17	31.34
												CDYWIR	N-Term(iTRAQ); C1(Methy)	1045.47	-	50.45
												ELTSELK	N-Term(iTRAQ); K7(iTRAQ)	1107.65	41.13	40.06
												TFVPGCQPGEFTLGNIKSYPGLT SYLVR	C6(Methy); K17(iTRAQ)	3234.65	13.14	-
VWF	P04275	von Willebrand factor	Up	2.07	1.93	48	48	145	20.69	3289	309.1	ILAGPAGDSNVVK	N-Term(iTRAQ); K13(iTRAQ)	1528.89	91.32	80.98
												ILTSDVFQDCNK	N-Term(iTRAQ); C10(Methy); K12(iTRAQ)	1716.85	79.63	66.35
												LVSVPYVGGNMEVNVYGAIMH EVR	N-Term(iTRAQ)	2777.42	77.32	36.9
												HGAGVAMDGQDVQLPLLK	N-Term(iTRAQ); M7(Oxida); K18(iTRAQ)	2153.17	73.97	40.37
												RLPGDIQVVPIGVGPNANVQEL ER	N-Term(iTRAQ)	2714.51	70.94	67.99
												EGGPSQIGDALGFAVR	N-Term(iTRAQ)	1717.90	70.56	54.76
												AEGLECTK	N-Term(iTRAQ); C6(Methy);	1184.59	67.19	62.66

												YAGSQVASTSEVLK	N-Term(iTRAQ); K14(iTRAQ)	1727.94	66.86	72.2
												FNHLGHIFTFTPQNNEFQLQLSPK	N-Term(iTRAQ); K24(iTRAQ)	3145.66	65.83	51.51
												FSEEACAVLTSPTFEACHR	N-Term(iTRAQ); C6(Methy);	2334.02	62.79	29.82
												GLYLETEAGYYK	N-Term(iTRAQ); K12(iTRAQ)	1694.89	61.31	72.75
												LLDLVFLLDGSSR	N-Term(iTRAQ)	1591.92	61.09	57.06
												VIVIPVGIGPHANLK	N-Term(iTRAQ); K15(iTRAQ)	1815.14	59.02	51.96
												LTGSCSYVLFQNK	N-Term(iTRAQ); C5(Methy);	1793.92	58.39	56.8
												EYAPGETVK	N-Term(iTRAQ); K9(iTRAQ)	1281.69	58.25	40.27
												EQAPNLVYMVTGNPASDEIKR	N-Term(iTRAQ); M9(Oxida); K20(iTRAQ)	2636.36	56.4	40.5
												IGWPNAPILIQDFETLPR	N-Term(iTRAQ)	2224.22	55.41	37.32
												YTLFQIFSK	N-Term(iTRAQ); K9(iTRAQ)	1434.82	55.1	61.14
												LSPVYAGK	N-Term(iTRAQ); K8(iTRAQ)	1122.68	54.44	38.39
												AHLLSLVDVMQR	N-Term(iTRAQ); M10(Oxida)	1541.86	51.97	55.26
												LSGEAYGFVAR	N-Term(iTRAQ)	1313.69	50.1	51.98
												GLWEQCQLLK	N-Term(iTRAQ); C6(Methy);	1551.83	49.54	44.52
												VSMPYASK	N-Term(iTRAQ); M3(Oxida);	1186.64	48.69	40.5
												LSEAEFEVLK	N-Term(iTRAQ); K10(iTRAQ)	1452.82	46.64	24.16
												SEVEVDIHYCQGK	N-Term(iTRAQ); C10(Methy); K13(iTRAQ)	1840.88	46.61	28.83
												QHSDPCALNPR	N-Term(iTRAQ); C6(Methy)	1427.66	44.4	31.03
												AFVLSSVDELEQQR	N-Term(iTRAQ)	1764.92	44.04	71.73
												HIVTFDGQNFK	N-Term(iTRAQ); K11(iTRAQ)	1593.86	42.78	29.08
												QTMVDSSCR	N-Term(iTRAQ); C8(Methy)	1216.53	42.7	-
												LTQVSVLQYGSITTIDVPWNVVPEK	N-Term(iTRAQ); K25(iTRAQ)	3074.71	41.21	-
												ICMDEDGNEK	N-Term(iTRAQ); C2(Methy); M3(Oxida); K10(iTRAQ)	1503.64	41.09	44.51
												GEYFWEK	N-Term(iTRAQ); K7(iTRAQ)	1246.64	41.04	49.86
												VTVFPIGIGDR	N-Term(iTRAQ)	1317.76	41.04	32.98
												VEDFGNAWK	N-Term(iTRAQ); K9(iTRAQ)	1353.71	40.49	53.49
												SKEFMEEVIQR	N-Term(iTRAQ); K2(iTRAQ);	1699.89	38.71	25.51
												RYPPGTSLSR	N-Term(iTRAQ)	1277.71	36.87	49.06
												GGQIMTLK	N-Term(iTRAQ); K8(iTRAQ)	1135.68	36.31	53.94
												CLPTACTIQLR	N-Term(iTRAQ); C1(Methy); C6(Methy)	1454.71	35.44	42.89
												VKEEVFIQQR	N-Term(iTRAQ); K2(iTRAQ)	1563.91	34.66	51.56
												EFMEEVIQR	N-Term(iTRAQ); M3(Oxida)	1340.66	29.33	19.2
												SGFTYVLHEGECCGR	N-Term(iTRAQ); C12(Methy); C13(Methy)	1893.79	28.6	14.57
												IQHTVTASVR	N-Term(iTRAQ)	1255.72	27.62	-
												AFVVDMMER	N-Term(iTRAQ)	1241.61	26.26	30.66
												GGQIMTLK	N-Term(iTRAQ); M5(Oxida);	1151.67	38.94	30.27
												ILVGNK	N-Term(iTRAQ); K6(iTRAQ)	931.62	32.33	-
												EAPDLVLQR	N-Term(iTRAQ)	1184.67	30.81	-
												APTCGLCEVAR	N-Term(iTRAQ); C4(Methy); C7(Methy)	1355.60	36.16	28.94
												CVALER	N-Term(iTRAQ); C1(Methy)	880.45	-	39.61
												YVQGLK	N-Term(iTRAQ); K6(iTRAQ)	995.61	-	32.56
RNASE3	P12724	Eosinophil cationic protein	Up	2.06	2.01	3	3	7	16.88	143	18.4	AQWFAIQHISLNPPR	N-Term(iTRAQ)	1922.05	58.88	32.49
												FYVVACDNR	N-Term(iTRAQ); C6(Methy)	1276.60	36.21	43.03
												FYVVACDNRDPR	N-Term(iTRAQ); C6(Methy)	1644.77	27.14	-
AZU1	P20160	Azurocidin	Up	2.06	2.87	4	4	9	22.71	176	26.9	QFPFLASIQNQGR	N-Term(iTRAQ)	1649.89	62.6	57.45
												CQVAGWGSQR	N-Term(iTRAQ); C1(Methy)	1281.58	34.86	39.96
												FVMTAASCFQSQNPGVSTVVLGAYDLR	N-Term(iTRAQ); C8(Methy)	3051.49	29.34	-
												GPDFFTR	N-Term(iTRAQ)	983.51	28.62	-
PRTN3	P24158	Myeloblastin	Up	2.02	3.06	3	3	24	17.19	532	27.8	TQEPTQQHFSVAQVFLNNYDAENK	N-Term(iTRAQ); K24(iTRAQ)	3096.52	84.39	45.56

												LVNVVLGAHNVR	N-Term(iTRAQ)	1434.87	61.73	50.67
												LFPDFFTR	N-Term(iTRAQ)	1186.64	31.63	-
CRISP3	P54108	Cysteine-rich secretory protein 3	Up	1.85	1.68	3	3	11	9.39	182	27.6	YEDLYSNCK	N-Term(iTRAQ); C8(Methy);	1468.67	58.6	44.65
												WANQCNYR	N-Term(iTRAQ); C5(Methy)	1244.54	24.54	-
												LTLTCK	N-Term(iTRAQ); C5(Methy);	1012.58	42.99	30.94
S100A9	P06702	Protein S100-A9	Up	1.79	1.99	10	10	892	68.42	23311	13.2	VIEHIMEDLDTNADK	N-Term(iTRAQ); M6(Oxida); K15(iTRAQ)	2047.02	115.61	89.12
												NIETIINTFHQYSVK	N-Term(iTRAQ); K15(iTRAQ)	2095.14	107.86	85.96
												LGHPDTLNQGEFK	N-Term(iTRAQ); K13(iTRAQ)	1743.93	91.91	63.76
												VIEHIMEDLDTNADK	M6(Oxida); K15(iTRAQ)	1902.92	83.31	54.18
												LGHPDTLNQGEFK	K13(iTRAQ)	1599.82	82.4	66.32
												VIEHIMEDLDTNADK	K15(iTRAQ)	1886.93	77.15	80.86
												QLSFEEFIMLMAR	N-Term(iTRAQ); M9(Oxida)	1774.90	74.37	72.87
												NIETIINTFHQYSVK	K15(iTRAQ)	1951.04	70.9	61.7
												VIEHIMEDLDTNADK	N-Term(iTRAQ); K15(iTRAQ)	2031.03	68.7	71.95
												LTWASHEK	K8(iTRAQ)	1115.60	68.09	-
												LTWASHEK	N-Term(iTRAQ); K8(iTRAQ)	1259.70	66.09	72.29
												QLSFEEFIMLMAR	N-Term(iTRAQ)	1758.91	62.56	63.95
												QLSFEEFIMLMAR	N-Term(iTRAQ); M9(Oxida);	1790.90	52.84	37.59
												DLQNFLK	N-Term(iTRAQ); K7(iTRAQ)	1165.68	48.99	49.78
												MSQLERNIETIINTFHQYSVK	N-Term(iTRAQ); M1(Oxida); K21(iTRAQ)	2855.50	48.08	24.56
												KDLQNFLK	N-Term(iTRAQ); K1(iTRAQ);	1437.88	47.94	48.19
												VIEHIMEDLDTNADKQLSFEEFIMLMAR	N-Term(iTRAQ); M6(Oxida); K15(iTRAQ); M24(Oxida); M26(Oxida)	3674.80	31.87	29.43
												KDLQNFLK	N-Term(iTRAQ); K8(iTRAQ)	1293.78	53.1	37.11
												MSQLER	N-Term(iTRAQ)	907.48	-	38.81
TCN1	P20061	Transcobalamin-1	Up	1.78	1.79	6	6	22	19.63	639	48.2	LVGIQIQTLMQK	N-Term(iTRAQ); K12(iTRAQ)	1660.01	93.28	74.96
												TYWELLSGGEPLSQGAGSYVVR	N-Term(iTRAQ)	2513.27	73.49	39
												GTSAVNVVLSLK	N-Term(iTRAQ); K12(iTRAQ)	1475.90	72.17	72.42
												NAEENLIYDYHLIDK	N-Term(iTRAQ); K15(iTRAQ)	2138.10	64.44	40.09
												LVGIQIQTLMQK	N-Term(iTRAQ); M10(Oxida); K12(iTRAQ)	1676.00	63.21	31
												NAEENLIYDYHLIDKLENK	N-Term(iTRAQ); K15(iTRAQ); K19(iTRAQ)	2766.47	53.89	28.48
												LSDVSSGELALIILALGVCR	N-Term(iTRAQ); C19(Methy)	2219.23	47.75	35.12
FCGBP	Q9Y6R7	IgGFc-binding protein	Up	1.77	1.75	36	36	96	12.23	2183	571.6	VTLQPYNVAQLQSSVDLSGSK	N-Term(iTRAQ); K21(iTRAQ)	2522.36	94.97	29.86
												LLISSLSESPASVSILSQADNTSK	N-Term(iTRAQ); K24(iTRAQ)	2735.50	88.54	43.5
												LDSLVAQQLQSK	N-Term(iTRAQ); K12(iTRAQ)	1617.94	87.64	72.93
												AISGLTIDGHAVGAK	N-Term(iTRAQ); K15(iTRAQ)	1697.98	85.6	84.59
												TEAVGQVHIFFQDGMVTLTPNK	N-Term(iTRAQ); M15(Oxida);	2736.43	85.08	62.24
												VAVIVSNDHAGK	N-Term(iTRAQ); K12(iTRAQ)	1497.86	80.83	81.99
												GEVGFVLVDNQR	N-Term(iTRAQ)	1476.81	76.57	54.87
												SLAAYTAACQAAGVAVKPWR	N-Term(iTRAQ); C9(Methy);	2368.25	75.13	51.49
												YDLAFVVASQATK	N-Term(iTRAQ); K13(iTRAQ)	1700.94	74.3	75.37
												ASQHGSDVVIETDFGLR	N-Term(iTRAQ)	1975.00	60.42	60.21
												LLFDGDAHLLMSIPSPFR	N-Term(iTRAQ); M11(Oxida)	2189.15	54.79	37.21
												AYSHSVSLTR	N-Term(iTRAQ)	1264.68	54.62	31.82
												VPAAYAASLCGLCGNYNQDPADDLK	N-Term(iTRAQ); C10(Methy); C13(Methy); K25(iTRAQ)	2949.33	54.46	78.37
												LTYNHGGITGSR	N-Term(iTRAQ)	1419.75	52.49	72.54
												LVDPQGPLK	N-Term(iTRAQ); K9(iTRAQ)	1254.76	51.97	-
												NMVLQTTK	N-Term(iTRAQ); K8(iTRAQ)	1222.70	50.99	35.19
												ISVAQGASK	N-Term(iTRAQ); K9(iTRAQ)	1148.69	50.42	53.3
												AIGYATAADCGR	N-Term(iTRAQ); C10(Methy)	1358.63	47.49	53.11
												ISVTQGASK	N-Term(iTRAQ); K9(iTRAQ)	1178.70	44.09	42.18
												CPGLQNTIPWYR	N-Term(iTRAQ); C1(Methy)	1637.80	42.14	-
												YYPLGEVFYPGPECER	N-Term(iTRAQ); C14(Methy)	2108.96	41.43	43.26

												RVSYVGLVTVR	N-Term(iTRAQ)	1392.84	41.15	49.04
												NMVLQTTK	N-Term(iTRAQ); M2(Oxida);	1238.70	40.66	38.79
												LLFDGDAHLLMSIPSPFR	N-Term(iTRAQ)	2173.16	39.52	42.61
												VSYVGLVTVR	N-Term(iTRAQ)	1236.74	39.26	30.79
												VAYDLVYYVR	N-Term(iTRAQ)	1404.76	39.06	32.34
												LCGMLTK	N-Term(iTRAQ); C2(Methy);	1099.59	38.86	40.49
												GGGQAANALAFGNSWQEETRPGCGATEPGDCPK	N-Term(iTRAQ); C23(Methy); C31(Methy); K33(iTRAQ)	3656.62	36.19	10.34
												VPAAYAGSLCGLCGNYNQDPADDLK	N-Term(iTRAQ); C10(Methy); C13(Methy); K25(iTRAQ)	2935.33	34.39	31.17
												AGCVAESTAVCR	N-Term(iTRAQ); C3(Methy);	1402.60	33.99	49.78
												EYPGQVLVDDVLQYLPFQAADGQVQVFR	N-Term(iTRAQ)	3338.72	30.97	-
												SVPGCEGVALVVAQTK	N-Term(iTRAQ); C5(Methy);	1892.02	29.82	20.99
												NTGREEFLTAFLQNYQLAYSK	N-Term(iTRAQ); K21(iTRAQ)	2781.45	26.99	40.29
												VLVENEHR	N-Term(iTRAQ)	1139.63	-	38.25
												CLLPGQSGPLCDALATYAAACQAAGATVHPWR	N-Term(iTRAQ); C1(Methy); C11(Methy); C21(Methy)	3494.61	-	15.25
												GNPAVSYVR	N-Term(iTRAQ)	1106.61	18.71	29.58
												LCGMLTK	N-Term(iTRAQ); C2(Methy); M4(Oxida); K7(iTRAQ)	1115.58	37.08	-
												LDPQGAVR	N-Term(iTRAQ)	999.57	25.47	-
												FYPAGDVLR	N-Term(iTRAQ)	1181.65	26.4	-
HIST1H2BK	O60814	Histone H2B type 1-K	Up	1.76	1.68	2	7	57	46.03	1511	13.9	AMGIMNSFVNDIFER	N-Term(iTRAQ)	1887.91	104.27	81.8
												AMGIMNSFVNDIFER	N-Term(iTRAQ); M5(Oxida)	1903.91	93.02	66.33
												QVHPDTGISSK	N-Term(iTRAQ); K11(iTRAQ)	1456.80	67.45	63.26
												LLLPGELAK	N-Term(iTRAQ); K9(iTRAQ)	1241.81	62.05	48.14
												AMGIMNSFVNDIFER	N-Term(iTRAQ); M2(Oxida); M5(Oxida)	1919.91	53.84	23.17
												KESYSVYVYK	N-Term(iTRAQ); K1(iTRAQ); K10(iTRAQ)	1697.95	51.91	43.25
												ESYSVYVYK	N-Term(iTRAQ); K9(iTRAQ)	1425.75	51.84	71.13
												EIQTAVR	N-Term(iTRAQ)	960.56	41.85	64.56
												LLLPGELAK	K9(iTRAQ)	1097.71	44.36	-
												LAHYNK	N-Term(iTRAQ); K6(iTRAQ)	1033.60	38.34	37.42
IGHD	P01880	Ig delta chain C	Up	1.76	1.69	10	10	39	42.71	765	42.2	SLWNAGTSVTCTLNHPSLPPQR	N-Term(iTRAQ); C11(Methy)	2569.27	89.58	50.68
												VPAPPSPQPATYTCVVSHEDSR	N-Term(iTRAQ); C14(Methy)	2528.20	79.76	42.66
												DSYYMTSSQLSTPLQQWR	N-Term(iTRAQ)	2335.12	73.7	48.88
												VPTGGVEEGLLER	N-Term(iTRAQ)	1499.82	60.92	50.74
												EPAAQAPVK	N-Term(iTRAQ); K9(iTRAQ)	1198.70	52.66	30.7
												ATFTCFVVGSDLK	N-Term(iTRAQ); C5(Methy);	1721.89	44.71	42.8
												TPECPSHTQPLGVYLLTPAVQDLWLR	N-Term(iTRAQ); C4(Methy)	3124.59	42.62	-
												APDVFPIISGCR	N-Term(iTRAQ); C11(Methy)	1464.74	36.23	37.2
												EVNTSGFAPARPPPQPGSTTFWAWSVLR	N-Term(iTRAQ)	3200.64	32.13	42.04
												RDSYYMTSSQLSTPLQQWR	N-Term(iTRAQ); M6(Oxida)	2507.21	21.35	26.61
MPO	P05164	Myeloperoxidase	Up	1.72	2.14	36	39	221	53.96	3938	83.8	IICDNTGITTVSK	N-Term(iTRAQ); C3(Methy);	1698.91	99.27	69.52
												NQINALTSFVDASMVYGSEEPLAR	N-Term(iTRAQ)	2756.37	96.03	27.84
												SLMFMQWGQLLDHDLDFTPEPAAR	N-Term(iTRAQ); M3(Oxida)	2978.42	84.8	46.12
												SLMFMQWGQLLDHDLDFTPEPAAR	N-Term(iTRAQ)	2962.44	75.82	65.23
												SLMFMQWGQLLDHDLDFTPEPAAR	N-Term(iTRAQ); M3(Oxida); M5(Oxida)	2994.44	71.07	32.68
												VGPLLACIIGTQFR	N-Term(iTRAQ); C7(Methy)	1677.94	63.22	49.9
												DGDRFWWENEGVFSMQQR	N-Term(iTRAQ); M15(Oxida)	2447.10	62.51	30.68
												VVLEGGIDPILR	N-Term(iTRAQ)	1424.86	61.66	60.64

												IVGAMVQIITYR	N-Term(iTRAQ)	1507.88	59.98	74.48
												LMEQYGTPNNIDIWMGGVSEPLKR	N-Term(iTRAQ); M15(Oxida); K23(iTRAQ)	3052.55	58.75	32.31
												DFVNCSTLPALNLASWR	N-Term(iTRAQ); C5(Methy)	2097.03	58.62	25.75
												FWWENEGVFSMQQR	N-Term(iTRAQ); M11(Oxida)	2003.92	58.1	44.58
												QALAQISLPR	N-Term(iTRAQ)	1240.75	56.83	-
												GLMATPAK	N-Term(iTRAQ); K8(iTRAQ)	1076.64	56.61	45.7
												DYLPLVLGPTAMR	N-Term(iTRAQ)	1589.88	56.27	38.36
												IGLDLPALNMQR	N-Term(iTRAQ)	1484.85	56.17	32.14
												QNQIAVDEIR		1185.62	55.62	39.16
												DYLPLVLGPTAMR	N-Term(iTRAQ); M12(Oxida)	1605.88	55.13	33.69
												IGLDLPALNMQR	N-Term(iTRAQ); M10(Oxida)	1500.83	54.73	40.19
												KIVGAMVQIITYR	N-Term(iTRAQ); K1(iTRAQ);	1796.07	53.52	45
												FPTDQLTPDQER	N-Term(iTRAQ)	1590.79	50.07	71
												FCGLPQPETVGQLGTVLR	N-Term(iTRAQ); C2(Methy)	2105.09	49.98	36.09
												IANVFTNAFR	N-Term(iTRAQ)	1296.72	48.93	54.08
												DHGLPGYNAWR	N-Term(iTRAQ)	1429.71	47.83	43.21
												GLMATPAK	N-Term(iTRAQ); M3(Oxida);	1092.63	46.9	40.09
												IANVFTNAFR		1152.62	45.76	28.91
												SSEMPELTSMHTLLLR	N-Term(iTRAQ); M4(Oxida)	2005.03	44.73	53.87
												YQPMEPNPR	N-Term(iTRAQ)	1275.63	44.51	32.83
												KLMEQYGTPNNIDIWMGGVSEPLK	M3(Oxida); K24(iTRAQ)	2880.44	44.37	50.57
												LMEQYGTPNNIDIWMGGVSEPLKR	N-Term(iTRAQ); M2(Oxida); M15(Oxida); K23(iTRAQ)	3068.54	44.24	18.99
												YQPMEPNPR	N-Term(iTRAQ); M4(Oxida)	1291.62	38.3	33.45
												WLPAEYEDGFSLPYGWTPGVK	N-Term(iTRAQ); K21(iTRAQ)	2700.36	37.68	28.25
												QNQIAVDEIR	N-Term(iTRAQ)	1329.73	36.32	55.39
												NQADCIPFFR	N-Term(iTRAQ); C5(Methy)	1400.66	33.27	48.29
												RFCGLPQPETVGQLGTVLR	N-Term(iTRAQ); C3(Methy)	2261.20	33.24	57.2
												YGHTLIQPFMFR	N-Term(iTRAQ)	1653.87	32.46	45.88
												IKNQADCIPFFR	N-Term(iTRAQ); K2(iTRAQ);	1785.94	32.34	15.8
												IVGAMVQIITYR	N-Term(iTRAQ); M5(Oxida)	1523.89	32.19	45.32
												SSEMPELTSMHTLLLR	N-Term(iTRAQ)	1989.02	31.08	22.89
												WLPAEYEDGFSLPYGWTPGVK	N-Term(iTRAQ); K21(iTRAQ)	2856.46	27.43	29.96
												KYLPTYR	N-Term(iTRAQ); K1(iTRAQ)	1228.73	23.93	-
												YGHTLIQPFMFR	N-Term(iTRAQ); M10(Oxida)	1669.87	21.34	28.95
												LMEQYGTPNNIDIWMGGVSEPLK	N-Term(iTRAQ); M2(Oxida); K23(iTRAQ)	2896.45	19.87	27.6
												IPCFLAGDTR	N-Term(iTRAQ); C3(Methy)	1282.64	44.67	28.79
												LATELK	N-Term(iTRAQ); K6(iTRAQ)	962.61	44.4	36.41
												NNIFMSNSYPR	N-Term(iTRAQ); M5(Oxida)	1502.72	29.53	30.07
												AVSNEIVR	N-Term(iTRAQ)	1031.60	28	31.07
												LFEQVMR	N-Term(iTRAQ); M6(Oxida)	1082.58	-	43.19
												RSPTLGASNR	N-Term(iTRAQ)	1202.67	-	37.36
												VFFASWR	N-Term(iTRAQ)	1056.58	25.23	37.36
												SPTLGASNR	N-Term(iTRAQ)	1046.57	-	33.09
												SSEMPELTSMHTLLLR	N-Term(iTRAQ); M4(Oxida);	2021.00	11.32	-
												VFFASWR		912.47	41.24	-
CD5L	O43866	CD5 antigen-like	Up	1.72	1.79	13	13	48	44.96	1235	38.1	CSGEEQSLEQCQHR	N-Term(iTRAQ); C1(Methy);	1869.75	80.58	76.68
												ELGCGAASGTPSGILYEPPAEK	N-Term(iTRAQ); C4(Methy);	2481.22	78.82	33.59
												EATLQDCPSGPWGK	N-Term(iTRAQ); C7(Methy);	1822.87	72.51	68.5
												GQWGTVCDDGWDIK	N-Term(iTRAQ); C7(Methy);	1913.88	56.99	56.23
												NTCNHDEDTWVECEDPFDLR	N-Term(iTRAQ); C3(Methy);	2674.04	50.45	58.93
												LVGGDNLCSGR	N-Term(iTRAQ); C8(Methy)	1280.62	46.34	39.51
												HQNQWYTVCQTGWSLR	N-Term(iTRAQ); C9(Methy)	2197.02	45.94	46.89
												FWGFHDCTHQEDVAVICSG	N-Term(iTRAQ); C7(Methy);	2386.98	44.43	-
												GQWGTVCDDGWDIKDVAVLCR	N-Term(iTRAQ); C7(Methy);	2716.26	33.09	37.09

												LEVLHK	N-Term(iTRAQ); K6(iTRAQ)	1026.65	43.41	33.51
												IWLDNVR	N-Term(iTRAQ)	1059.60	35.27	38.95
												DVAVLCR	N-Term(iTRAQ); C6(Methy)	965.50	35.23	41.94
												AVLTQK	N-Term(iTRAQ); K6(iTRAQ)	947.61	39.83	38.39
GALNS	P34059	N-acetylgalactosamine-6-sulfatase	Up	1.71	1.78	2	2	4	4.21	54	58.0	GDTLMAATLGQHK	N-Term(iTRAQ); M5(Oxida); K13(iTRAQ)	1646.88	40.38	25.14
												LPLIFHLGR	N-Term(iTRAQ)	1209.76	23.83	18.51
S100A8	P05109	Protein S100-A8	Up	1.70	1.88	12	12	1112	88.17	24579	10.8	ALNSIIDVYHK	N-Term(iTRAQ); K11(iTRAQ)	1560.90	101.84	91.01
												ELDINTDGAVNFQEFLILVIK	N-Term(iTRAQ); K21(iTRAQ)	2679.48	79.29	84.48
												MLTELEKALNSIIDVYHK	N-Term(iTRAQ); M1(Oxida); K18(iTRAQ)	2421.33	67.6	59.28
												ALNSIIDVYHKYSLIK	N-Term(iTRAQ); K16(iTRAQ)	2165.26	64.91	41.56
												LLETECPQYIR	C6(Methy)	1410.68	63.64	63.63
												MLTELEK	N-Term(iTRAQ); K7(iTRAQ)	1151.65	53.94	53.61
												GADVWFK	N-Term(iTRAQ); K7(iTRAQ)	1110.61	51.58	50.49
												KLLETECPQYIR	N-Term(iTRAQ); K1(iTRAQ);	1826.98	48.81	56.87
												LLETECPQYIR	N-Term(iTRAQ); C6(Methy)	1554.78	47.12	51.97
												GNFHAVYR	N-Term(iTRAQ)	1107.58	43.42	65.73
												ALNSIIDVYHK	K11(iTRAQ)	1416.80	43.25	25.6
												GNFHAVYRDDLK	N-Term(iTRAQ); K12(iTRAQ)	1722.92	39.95	44.94
												MLTELEK	N-Term(iTRAQ); M1(Oxida);	1167.65	37.86	41
												ELDINTDGAVNFQEFLILVIKMGVAAHK	N-Term(iTRAQ); K21(iTRAQ); M22(Oxida); K28(iTRAQ)	3533.93	36.23	28.73
												YSLIKGNFHAVYR	N-Term(iTRAQ)	1711.94	33.97	-
												KLLETECPQYIR	N-Term(iTRAQ); C7(Methy)	1682.87	23.42	24.04
												MLTELEK	K7(iTRAQ)	1007.55	42.78	48.76
												MLTELEK	M1(Oxida); K7(iTRAQ)	1023.55	42.72	39.22
												GNFHAVYR		963.48	-	35.57
DEFA1	P59665	Neutrophil defensin 1	Up	1.68	1.78	3	3	14	20.21	192	10.2	YGTCIYQGR	N-Term(iTRAQ); C4(Methy)	1250.58	39.37	29.54
												RYGTCIYQGR	C5(Methy)	1262.58	33.71	-
												RYGTCIYQGR	N-Term(iTRAQ); C5(Methy)	1406.68	26.39	31.08
												IPACIAGER	C4(Methy)	975.48	42.4	42.97
												IPACIAGER	N-Term(iTRAQ); C4(Methy)	1119.57	26.48	38.95
HIST1H4A	P62805	Histone H4	Up	1.67	1.79	10	10	159	58.25	3511	11.4	TVTAMDVVYALK	N-Term(iTRAQ); M5(Oxida); K12(iTRAQ)	1614.90	84.96	81.93
												DAVTYTEHAK	K10(iTRAQ)	1278.65	80.82	55.01
												TVTAMDVVYALK	N-Term(iTRAQ); K12(iTRAQ)	1598.90	79.49	98.5
												DAVTYTEHAK	N-Term(iTRAQ); K10(iTRAQ)	1422.75	77.57	79.3
												TVTAMDVVYALKR	N-Term(iTRAQ); M5(Oxida); K12(iTRAQ)	1771.00	73.65	54.2
												TVTAMDVVYALKR	N-Term(iTRAQ); K12(iTRAQ)	1755.01	59.09	33.47
												GVLKVFLENVIR	N-Term(iTRAQ)	1530.95	52.84	58.12
												DNIQGITKPAIR	N-Term(iTRAQ); K8(iTRAQ)	1613.96	51.45	56.47
												GVLKVFLENVIR	N-Term(iTRAQ); K4(iTRAQ)	1675.05	49.56	47.81
												DNIQGITKPAIR	K8(iTRAQ)	1469.86	47.46	40.1
												ISGLIYEETR	N-Term(iTRAQ)	1324.73	46.04	53.87
												KTVTAMDVVYALK	N-Term(iTRAQ); K1(iTRAQ); M6(Oxida); K13(iTRAQ)	1887.10	45.41	45.8
												DAVTYTEHAKR	N-Term(iTRAQ); K10(iTRAQ)	1578.85	41.73	38.05
												VFLENVIR	N-Term(iTRAQ)	1133.68	31.62	41.82
												DNIQGITKPAIRR	N-Term(iTRAQ); K8(iTRAQ)	1770.06	16.13	14.26
												VFLENVIR		989.58	40.27	28.91
ADIPOQ	Q15848	Adiponectin	Up	1.67	1.67	4	4	31	23.77	637	26.4	SAFSVGLETYVTIPNMPIR	N-Term(iTRAQ)	2239.19	73.86	30.89
												IFYNQQNHYDGSTGK	N-Term(iTRAQ); K15(iTRAQ)	2060.01	64.69	53.37
												GDIGETGVPGAEGPR	N-Term(iTRAQ)	1555.78	55.63	52.01
												GDPGLIGPK	N-Term(iTRAQ); K9(iTRAQ)	1141.68	43.23	30.8
												SAFSVGLETYVTIPNMPIR	N-Term(iTRAQ); M16(Oxida)	2255.18	22.27	-
SHBG	P04278	Sex hormone-binding	Up	1.61	1.73	12	12	54	38.31	1299	43.8	DIPQPHAEPWAFSLDLGLK	N-Term(iTRAQ); K19(iTRAQ)	2422.31	81.09	63.92

		globulin										VVLSSGSGPGLDLPLVLGLPLQLK	N-Term(iTRAQ); K24(iTRAQ)	2660.61	79.55	33.83
												QAEISASAPTSLR	N-Term(iTRAQ)	1474.80	69.07	60.96
												TWDPEGVIFYGDTNPK	N-Term(iTRAQ); K16(iTRAQ)	2127.06	63.8	30.78
												ALALPPLGLAPLLNLWAKPQGR	N-Term(iTRAQ); K18(iTRAQ)	2597.59	60.69	31.56
												QVSGPLTSK	N-Term(iTRAQ); K9(iTRAQ)	1204.71	55.12	52.32
												VVLSQGSK	N-Term(iTRAQ); K8(iTRAQ)	1105.69	53.55	44.03
												IALGGLLFPASNLR	N-Term(iTRAQ)	1585.96	53.53	46.46
												DDWFMLGLR	N-Term(iTRAQ)	1296.65	42.79	42.58
												LPLVPALDGCLR	N-Term(iTRAQ); C10(Methy)	1456.82	35.1	41.45
												TWDPEGVIFYGDTNPKDDWFMLGLR	N-Term(iTRAQ); K16(iTRAQ)	3260.59	26.68	-
												TSSSFEVR	N-Term(iTRAQ)	1056.54	33.09	-
												DDWFMLGLR	N-Term(iTRAQ); M5(Oxida)	1312.65	-	22.61
CTSG	P08311	Cathepsin G	Up	1.58	1.63	6	6	29	31.37	655	28.8	GDSGGPLLCNNVAHGIVSYGK	N-Term(iTRAQ); C9(Methy);	2392.20	76.72	77.15
												TIQNDIMLLQLSR	N-Term(iTRAQ)	1688.95	73.87	47.6
												SSGVPPEVFTR	N-Term(iTRAQ)	1319.71	54.35	54.92
												AQEGLRPGTLCTVAGWGR	N-Term(iTRAQ); C11(Methy)	2062.05	49.44	56.49
												VSSFLPWIR	N-Term(iTRAQ)	1248.72	34.01	37.84
												VSSFLPWIR		1104.62	44.21	32.49
												IFGSYDPR	N-Term(iTRAQ)	1098.57	32.88	-
VCP	P55072	Transitional endoplasmic reticulum ATPase	Up	1.56	1.64	4	4	8	8.31	194	89.3	ELQELVQYPVEHPDK	N-Term(iTRAQ); K15(iTRAQ)	2112.12	65.43	64.06
												ETVVEVPQVTWEDIGGLEDVKR	N-Term(iTRAQ); K21(iTRAQ)	2786.49	61.6	38.77
												IVSQLLTLMDGLK	N-Term(iTRAQ); K13(iTRAQ)	1719.03	30.6	28.51
												NAPAIIFIDELDAIAPK	N-Term(iTRAQ); K17(iTRAQ)	2099.20	23.59	24.2
FCGR3A	P08637	Low affinity immunoglobulin gamma Fc region receptor III	Up	1.56	2.12	3	3	11	11.42	203	29.1	VLEKDSVTLK	N-Term(iTRAQ); K4(iTRAQ); K10(iTRAQ)	1563.97	54.28	62.23
												AVVFLEPQWYR	N-Term(iTRAQ)	1551.85	40.49	52.99
												EEDPIHLR	N-Term(iTRAQ)	1152.61	26.14	-
HIST1H2AH	Q96KK5	Histone H2A type 1-H	Up	1.51	1.62	4	4	44	50.00	1040	13.9	VGAGAPVYLAAVLEYLTAEILELAGNAAR	N-Term(iTRAQ)	3059.69	102.08	46.72
												VTIAQGGVLPNIQAVLLPK	N-Term(iTRAQ); K19(iTRAQ)	2219.36	48.64	72.73
												AGLQFPVGR	N-Term(iTRAQ)	1088.63	45.1	53.01
												HLQLAIR	N-Term(iTRAQ)	994.63	21.41	38.21
LYZ	P61626	Lysozyme C	Up	1.48	1.74	6	6	34	50.68	680	16.5	TPGAVNACHLSCSALLQDNIADAVACAK	N-Term(iTRAQ); C8(Methy); C12(Methy); C26(Methy); K28(iTRAQ)	3182.48	83.74	23
												GISLANWMCLAK	N-Term(iTRAQ); C9(Methy);	1640.86	74.13	81.96
												STDYGIFQINSR		1400.68	66.25	68.14
												STDYGIFQINSR	N-Term(iTRAQ)	1544.78	58.93	50.12
												GISLANWMCLAK	N-Term(iTRAQ); M8(Oxida); C9(Methy); K12(iTRAQ)	1656.85	43.18	21.74
												WESGYNTR	N-Term(iTRAQ)	1156.55	27.74	34.5
												QYVQGCGV	N-Term(iTRAQ); C6(Methy)	1043.48	39.37	-
												LGMDGYR	N-Term(iTRAQ)	955.48	26.2	35.93
LDHA	P00338	L-lactate dehydrogenase A chain	Up	1.47	1.72	17	18	45	55.72	1076	36.7	QVVESAYEVIK	N-Term(iTRAQ); K11(iTRAQ)	1552.88	85.2	91.18
												DLADELALVDVIEDKLK	N-Term(iTRAQ); K15(iTRAQ); K17(iTRAQ)	2331.34	80.21	72.5
												DLADELALVDVIEDK	N-Term(iTRAQ); K15(iTRAQ)	1946.06	67.88	32.57
												GYTSWAIGLSVADLAESIMK	N-Term(iTRAQ); K20(iTRAQ)	2400.29	65.6	34.14
												DQLIYNLLKEEQTPQNK	N-Term(iTRAQ); K9(iTRAQ); K17(iTRAQ)	2506.39	63.36	45.24
												SADTLWGIQK	N-Term(iTRAQ); K10(iTRAQ)	1406.79	63.12	65.91
												DYNVTANSK	N-Term(iTRAQ); K9(iTRAQ)	1299.68	62.63	63.14
												DQLIYNLLK	N-Term(iTRAQ); K9(iTRAQ)	1407.84	54.34	53.61
												VTLTSEEEAR	N-Term(iTRAQ)	1278.67	48.65	54.04
												FIIPNVVK	N-Term(iTRAQ); K8(iTRAQ)	1217.79	41.64	30.57
												GEMMDLQHGSLFLR	N-Term(iTRAQ); M3(Oxida)	1793.88	35.77	25.6
												VIGSGCNLDSAR	N-Term(iTRAQ); C6(Methy)	1381.66	31.36	39.77

												LVIITAGAR	N-Term(iTRAQ)	1057.69	28.04	46.41
												VHPVSTMIK	N-Term(iTRAQ); M7(Oxida);	1315.76	26.63	-
												GLYGIKDDVFLSVPCILGQNGIS DLVK	N-Term(iTRAQ); K6(iTRAQ); C15(Methy); K27(iTRAQ)	3341.80	18.1	-
												NVNIFK	N-Term(iTRAQ); K6(iTRAQ)	1022.62	37.16	35.25
												GLYGIK	N-Term(iTRAQ); K6(iTRAQ)	938.59	28.17	38.37
												YSPNCK	N-Term(iTRAQ); C5(Methy);	1045.50	37.68	-
PGLYRP1	O75594	Peptidoglycan recognition protein 1	Up	1.46	2.39	3	3	12	25.51	260	21.7	AAQGLLACGVAQGALR	N-Term(iTRAQ); C8(Methy)	1688.91	64.53	92.85
												TLSPGNQLYHLIQNWPHYR	N-Term(iTRAQ)	2481.30	52.74	29.72
												ALASECAQHLSLPLR	N-Term(iTRAQ); C6(Methy)	1798.94	26.14	21.71
MIF	P14174	Macrophage migration inhibitory factor	Up	1.46	1.75	2	2	7	17.39	102	12.5	PMFIVNTNVPR	N-Term(iTRAQ); M2(Oxida)	1447.78	42.41	17.41
												PMFIVNTNVPR	N-Term(iTRAQ)	1431.79	40.88	36.55
												LLCGLLAER	N-Term(iTRAQ); C3(Methy)	1177.66	24.23	44.71
IGLL1	P15814	Immunoglobulin lambda-like polypeptide	Up	1.45	1.68	2	2	4	7.51	23	22.9	RSYSCQVMHEGSTVEK	C5(Methy); K16(iTRAQ)	2030.92	23.44	-
												SYSCQVMHEGSTVEK	N-Term(iTRAQ); C4(Methy);	2018.94	21.65	-
ALPL	P05186	Alkaline phosphatase, tissue-nonspecific isozyme	Up	1.40	1.65	7	7	18	14.31	342	57.3	ANEGTVGVSAATER	N-Term(iTRAQ)	1505.76	68.77	93.7
												QALHEAVEMDR	N-Term(iTRAQ); M9(Oxida)	1458.71	46.51	31.39
												DIAYQLMHNIR	N-Term(iTRAQ); M7(Oxida)	1533.80	45.96	26.19
												LNTNVAK	N-Term(iTRAQ); K7(iTRAQ)	1047.64	45.23	45.85
												GFFLLVEGGR	N-Term(iTRAQ)	1238.70	38.77	43.98
												LEMDKFPFVALSK	N-Term(iTRAQ); M3(Oxida); K5(iTRAQ); K13(iTRAQ)	1973.13	35.28	-
												SVGIVTTTR	N-Term(iTRAQ)	1077.64	32.44	49.03
												QALHEAVEMDR	N-Term(iTRAQ)	1442.71	27.98	23.65
												DIAYQLMHNIR	N-Term(iTRAQ)	1517.80	-	29.64
AP3B2	Q13367	AP-3 complex subunit beta-2	Up	1.40	2.34	2	2	7	2.13	29	119.0	DQLIEVIEK	N-Term(iTRAQ); K9(iTRAQ)	1374.81	21.66	31.28
												KPYVMDPDHRLLLR	N-Term(iTRAQ)	1897.06	-	23.54
												KPYVMDPDHRLLLR	N-Term(iTRAQ); K1(iTRAQ);	2057.15	14.16	-
S100A6	P06703	Protein S100-A6	Up	1.39	2.03	4	4	9	47.78	126	10.2	LQDAEIAR	N-Term(iTRAQ)	1059.59	41.48	50.07
												DQEVNFQEYVTFLGALALIYNE ALKG	N-Term(iTRAQ); K25(iTRAQ)	3233.72	40.52	-
												NKDQEVNFQEYVTFLGALALIY NEALK	N-Term(iTRAQ); K2(iTRAQ); K27(iTRAQ)	3562.91	21.21	29.28
												LMEDLDR	N-Term(iTRAQ); M2(Oxida)	1051.52	29.23	-

[a]Accession No. means accession number of protein in the UniProt/Swiss-Prot database.

[b]Protein with ratio > mean + 1SD is defined as up-regulated.

[c]The value was obtained by normalized value of CPPE patients divided by UPPE patients in experiment 1.

[d]The value was obtained by normalized value of CPPE patients divided by UPPE patients in experiment 2.

UPPE: uncomplicated parapneumonic effusion; CPPE: complicated parapneumonic effusion

Supplementary Table 3. List of 35 down-regulated proteins in CPPE compared with UPPE

Gene Name	Accession No.[a]	Protein Name	Down-regulated[b]	Exp 1 (Fold)[c]	Exp 2 (Fold)[d]	Number of unique peptides	Number of total peptides	Number of total spectra	Sequence coverage (%)	Mascot score	MW (KDa)	Peptide sequence	Modification	Calculated MH+	IonScore (HCD)	IonScore (CID)
APOC3	P02656	Apolipoprotein C-III	Down	0.14	0.19	6	6	84	55.56	2824	10.8	DALSSVQESQVAQQAR	N-Term(iTRAQ)	1860.96	119.25	97.78
												DALSSVQESQVAQQAR		1716.85	104.85	81.49
												GWVTDGFSSLK	N-Term(iTRAQ); K11(iTRAQ)	1484.81	68.78	70.46
												DYWSTVK	N-Term(iTRAQ); K7(iTRAQ)	1186.64	41.84	47.66
												GWVTDGFSSLKDYWSTVK	N-Term(iTRAQ); K18(iTRAQ)	2364.21	30.98	26.66
												FSEFWDLDPEVRPTSAVAA	N-Term(iTRAQ)	2281.13	19.76	-
												DKFSEFWDLDPEVRPTSAVAA	N-Term(iTRAQ); K2(iTRAQ)	2668.35	18.79	22.57
VNN1	O95497	Pantetheinase	Down	0.29	0.37	8	8	25	18.91	704	57.0	LFSLKPTSGPVLTVTLFGR	N-Term(iTRAQ); K5(iTRAQ)	2321.39	84.67	70.47
												LFSLKPTSGPVLTVTLFGR	K5(iTRAQ)	2177.28	71.97	67.75
												GTVFFDEFTFVK	N-Term(iTRAQ); K12(iTRAQ)	1724.92	62.94	43.94
												YQYNTDVVFDSQGK	N-Term(iTRAQ); K14(iTRAQ)	1951.97	57.05	43.04
												VNFLASNIHYPSK	N-Term(iTRAQ); K13(iTRAQ)	1777.99	50.89	29.61
												EEALALMNR	N-Term(iTRAQ)	1190.63	45.60	51.87
												EEALALMNR	N-Term(iTRAQ); M7(Oxida)	1206.63	44.63	47.55
												TTNLNTCGDSAETASTR	N-Term(iTRAQ); C7(Methy)	1931.85	-	26.05
												LSCLAK	N-Term(iTRAQ); C3(Methy);	968.55	40.06	38.38
												AFHYDMK	N-Term(iTRAQ); M6(Oxida);	1215.61	36.91	-
EPX	P11678	Eosinophil peroxidase	Down	0.29	0.40	5	8	42	14.97	677	81.0	ASAPNSHVPLSSAFFASWR	N-Term(iTRAQ)	2176.11	59.35	30.29
												GLMATPAK	N-Term(iTRAQ); K8(iTRAQ)	1076.64	56.61	45.70
												DHGLPGYNAWR	N-Term(iTRAQ)	1429.71	47.83	43.21
												GLMATPAK	N-Term(iTRAQ); M3(Oxida);	1092.63	46.90	40.09
												KFLNLYGTPDNIDIWIGAIAEPLL PGAR	N-Term(iTRAQ); K1(iTRAQ)	3355.88	42.55	19.33
												VANVFTLAFR	N-Term(iTRAQ)	1281.75	37.88	28.80
												KIMGAMVQIITYR	N-Term(iTRAQ); K1(iTRAQ)	1812.05	29.93	43.24
												IPCFLAGDTR	N-Term(iTRAQ); C3(Methy)	1282.64	44.67	28.79
												AVSNQIVR	N-Term(iTRAQ)	1030.61	37.53	31.00
CLC	Q05315	Galectin-10	Down	0.31	0.36	3	3	8	14.08	125	16.4	FNVSYLK	N-Term(iTRAQ); K7(iTRAQ)	1158.68	53.93	39.09
												IKPEAVK	N-Term(iTRAQ); K2(iTRAQ);	1216.80	38.25	14.89
												MVQVWR	N-Term(iTRAQ)	962.54	-	34.57
APOC4	P55056	Apolipoprotein C-IV	Down	0.32	0.35	5	5	31	29.13	670	14.5	GFMQTYYDDHLR	N-Term(iTRAQ); M3(Oxida)	1705.79	78.83	50.01
												GFMQTYYDDHLR	N-Term(iTRAQ)	1689.78	64.51	45.62
												MKELLETVVNR	N-Term(iTRAQ); M1(Oxida);	1635.94	55.19	34.58
												ELLETVVNR	N-Term(iTRAQ)	1216.70	48.41	38.80
												AWFLESK	N-Term(iTRAQ); K7(iTRAQ)	1168.66	42.21	43.57
												DLGPLTK	N-Term(iTRAQ); K7(iTRAQ)	1031.64	40.88	35.65
APOC1	P02654	Apolipoprotein C-I	Down	0.32	0.36	6	6	50	37.35	968	9.3	EFGNTLEDK	N-Term(iTRAQ); K9(iTRAQ)	1340.69	72.43	59.82
												EWFSETFQK	N-Term(iTRAQ); K9(iTRAQ)	1489.76	62.59	63.55
												QSELSAK	N-Term(iTRAQ); K7(iTRAQ)	1050.60	55.40	37.42
												LKEFGNTLEDK	N-Term(iTRAQ); K11(iTRAQ)	1581.88	54.10	50.92
												LKEFGNTLEDK	N-Term(iTRAQ); K2(iTRAQ); K11(iTRAQ)	1725.98	46.98	58.94
												IKQSELSAK	N-Term(iTRAQ); K2(iTRAQ);	1435.89	30.17	44.15
												MREWFSETFQK	N-Term(iTRAQ); M1(Oxida); K11(iTRAQ)	1792.90	34.04	27.39
SPP2	Q13103	Secreted phosphoprotein 24	Down	0.35	0.59	2	2	5	9.95	160	24.3	DALSASVVK	N-Term(iTRAQ); K9(iTRAQ)	1177.70	67.43	54.15
												VSAQQVQGVHAR	N-Term(iTRAQ)	1423.79	62.26	52.55
APOC2	P02655	Apolipoprotein C-II	Down	0.39	0.36	4	4	105	49.5	2375	11.3	ESLSSYWESAK	N-Term(iTRAQ); K11(iTRAQ)	1574.80	87.18	85.86
												STAAMSTYTGIFTDQVLSVLK	N-Term(iTRAQ); K21(iTRAQ)	2521.35	80.29	115.55
												STAAMSTYTGIFTDQVLSVLK	N-Term(iTRAQ); M5(Oxida); K21(iTRAQ)	2537.35	74.53	31.35
												TYLPAVDEK	N-Term(iTRAQ); K9(iTRAQ)	1323.74	73.80	48.44
												TAAQNLYEK	N-Term(iTRAQ); K9(iTRAQ)	1325.73	58.22	57.70
												ESLSSYWESAK	K11(iTRAQ)	1430.71	56.11	41.91

												TYLPAVDEK	K9(iTRAQ)	1179.64	45.89	38.31
												STAAMSTYTGIFTDQVLSVLK	K21(iTRAQ)	2377.25	21.31	-
TF	P02787	Serotransferrin OS	Down	0.42	0.45	59	59	1523	74.5	33659	77.0	IECVSAETTEDCIAK	N-Term(iTRAQ); C3(Methy); C12(Methy); K15(iTRAQ)	1991.90	104.56	96.91
												FDEFFSEGCAPGSK	N-Term(iTRAQ); C9(Methy);	1854.84	93.08	104.38
												HSTIFENLANK	K11(iTRAQ)	1417.76	90.58	66.05
												SASDLTWDNLK	N-Term(iTRAQ); K11(iTRAQ)	1537.81	90.52	76.43
												LCMGSGLNLCEPNNK	N-Term(iTRAQ); C2(Methy); C10(Methy); K15(iTRAQ)	1972.90	88.94	93.71
												HSTIFENLANK	N-Term(iTRAQ); K11(iTRAQ)	1561.86	86.79	67.38
												KCSTSSLLEACTFR	N-Term(iTRAQ); C2(Methy);	1781.82	84.88	42.91
												LCMGSGLNLCEPNNK	N-Term(iTRAQ); C2(Methy); M3(Oxida); C10(Methy); K15(iTRAQ)	1988.89	84.81	71.01
												EDPQTFYYAVAVVK	N-Term(iTRAQ); K14(iTRAQ)	1918.03	84.55	93.90
												HQTVPQNTGGK	N-Term(iTRAQ); K11(iTRAQ)	1454.80	84.24	53.42
												SAGWNIPIGLLYCDLPEPR	N-Term(iTRAQ); C13(Methy)	2304.16	83.68	40.39
												DGAGDVAFVKHSTIFENLANK	N-Term(iTRAQ); K21(iTRAQ)	2521.34	83.05	53.51
												SVIPSDGPSVACVK	N-Term(iTRAQ); C12(Methy); K14(iTRAQ)	1692.89	82.57	75.42
												IMNGEADAMSLDGGFVYIAGK	N-Term(iTRAQ); K21(iTRAQ)	2447.22	81.38	70.24
												SVIPSDGPSVACVK	C12(Methy); K14(iTRAQ)	1548.79	80.43	33.68
												AIAANEADAVTLDAGLVYDAYLAPNNLKPVVAEFYGSK	N-Term(iTRAQ); K28(iTRAQ); K38(iTRAQ)	4386.33	79.12	14.92
												MYLGYEYVTAIR	N-Term(iTRAQ); M1(Oxida)	1638.84	78.01	67.38
												WCAVSEHEATK	N-Term(iTRAQ); C2(Methy);	1594.76	76.16	79.48
												ADRDQYELLCLDNTR	N-Term(iTRAQ); C10(Methy)	2014.95	74.79	58.60
												CDEWSVNSVGK	N-Term(iTRAQ); C1(Methy);	1557.73	74.57	81.93
												EDLIWELLNQAQEHFGK	N-Term(iTRAQ); K17(iTRAQ)	2358.23	74.30	65.01
												CSTSSLLEACTFR	N-Term(iTRAQ); C1(Methy);	1653.73	74.05	84.97
												EFQLFSSPHGK	N-Term(iTRAQ); K11(iTRAQ)	1564.84	67.28	85.44
												NLNEKDYELLCLDGTR	N-Term(iTRAQ); K5(iTRAQ);	2230.11	66.87	55.47
												FDEFFSEGCAPGSK	C9(Methy); K14(iTRAQ)	1710.73	65.80	80.48
												DGAGDVAFVK	N-Term(iTRAQ); K10(iTRAQ)	1266.70	65.24	62.26
												NLNEKDYELLCLDGTR	N-Term(iTRAQ); C11(Methy)	2086.01	65.08	56.19
												FDEFFSEGCAPGSKK	N-Term(iTRAQ); C9(Methy);	1982.93	63.25	50.95
												YLGEEYVKAVGNLR	N-Term(iTRAQ)	1754.96	62.77	68.32
												DCHLAQVPSHTVVAR	N-Term(iTRAQ); C2(Methy)	1822.92	62.19	42.45
												KDSGFQMNQLR	N-Term(iTRAQ); M7(Oxida)	1483.74	61.46	36.10
												IMNGEADAMSLDGGFVYIAGK	N-Term(iTRAQ); M2(Oxida); M9(Oxida); K21(iTRAQ)	2479.21	60.54	35.20
												QQQHLFGSNVTDCSGNFCLFR	N-Term(iTRAQ); C13(Methy); C18(Methy)	2637.15	60.14	54.39
												EGTCPEAPTDECKPVK	N-Term(iTRAQ); C4(Methy); C12(Methy); K16(iTRAQ)	2083.94	59.45	46.21
												TAGWNIPMGLLYNK	N-Term(iTRAQ); K14(iTRAQ)	1866.02	58.71	74.58
												DLLFRDDTVCLAK	N-Term(iTRAQ); C10(Methy); K13(iTRAQ)	1842.98	58.26	63.47
												KDSGFQMNQLR	N-Term(iTRAQ); K1(iTRAQ);	1627.85	57.97	47.60
												DDTVCLAK	N-Term(iTRAQ); C5(Methy);	1198.61	57.46	60.55
												DCHLAQVPSHTVVAR	C2(Methy)	1678.82	57.29	43.73
												DSGFQMNQLR	N-Term(iTRAQ); M6(Oxida)	1355.65	57.18	26.22
												APNHAVVTR	N-Term(iTRAQ)	1108.64	57.01	54.16
												SMGGKEDLIWELLNQAQEHFGK	N-Term(iTRAQ); M2(Oxida);	2834.44	56.15	42.11
												TAGWNIPMGLLYNK	N-Term(iTRAQ); M8(Oxida); K14(iTRAQ)	1882.02	56.06	53.65
												EDPQTFYYAVAVVKK	N-Term(iTRAQ); K15(iTRAQ)	2046.12	55.86	53.76
												CLVEKGDVAFVK	N-Term(iTRAQ); C1(Methy);	1641.90	54.61	50.07
												GDVAFVKHQTVPQNTGGK	N-Term(iTRAQ); K18(iTRAQ)	2171.18	54.58	49.95

												EDLIWELLNQAQEHFGK	K17(iTRAQ)	2214.13	54.25	38.41
												YLGEEYVK	N-Term(iTRAQ); K8(iTRAQ)	1288.70	53.61	54.44
												TAGWNIPMGLLYNK	K14(iTRAQ)	1721.92	52.68	41.42
												NTYEKYLGEEYVK	N-Term(iTRAQ); K13(iTRAQ)	1924.00	51.93	38.37
												NPDPWAK	N-Term(iTRAQ); K7(iTRAQ)	1115.61	51.89	44.85
												EGYYGYTGAFR	N-Term(iTRAQ)	1427.67	51.46	58.03
												DSGFQMNQLR		1195.55	51.29	54.99
												DYELLCLDGTR	N-Term(iTRAQ); C6(Methy)	1487.70	50.61	57.26
												DLLFKDSAHGFLK	N-Term(iTRAQ); K13(iTRAQ)	1779.01	49.95	48.30
												MDAKMYLGYEYVTAIR	N-Term(iTRAQ); M5(Oxida)	2084.04	49.93	55.31
												MDAKMYLGYEYVTAIR	N-Term(iTRAQ); K4(iTRAQ);	2228.14	49.82	34.45
												DSAHGFLK	N-Term(iTRAQ); K8(iTRAQ)	1162.65	48.76	47.49
												MDAKMYLGYEYVTAIR	N-Term(iTRAQ); M1(Oxida); M5(Oxida)	2100.03	48.48	50.57
												DQYELLCLDNTR	N-Term(iTRAQ); C7(Methy)	1672.77	47.96	52.90
												MDAKMYLGYEYVTAIR	N-Term(iTRAQ); M1(Oxida); K4(iTRAQ); M5(Oxida)	2244.13	47.57	31.61
												KPVDEYK	N-Term(iTRAQ); K1(iTRAQ);	1310.77	47.43	46.36
												EFQLFSSPHGK	K11(iTRAQ)	1420.74	47.01	51.37
												IMNGEADAMSLDGGFVYIAGK	N-Term(iTRAQ); M2(Oxida); K21(iTRAQ)	2463.21	46.83	25.11
												MYLGYEYVTAIR		1478.73	46.21	60.57
												MYLGYEYVTAIR	N-Term(iTRAQ)	1622.83	45.88	40.88
												LCMGSGLNLCEPNNK	C2(Methy); C10(Methy); K15(iTRAQ)	1828.80	45.65	36.81
												KPVDEYKDCHLAQVPSHTVVA	N-Term(iTRAQ); K7(iTRAQ);	2826.46	45.11	-
												SETKDLLFR	N-Term(iTRAQ)	1252.70	44.42	-
												EDLIWELLNQAQEHFGKDK	N-Term(iTRAQ); K17(iTRAQ); K19(iTRAQ)	2745.47	44.04	29.41
												KPVEEYANCHLAR	N-Term(iTRAQ); K1(iTRAQ);	1863.93	42.74	51.59
												KASYLDCIR	N-Term(iTRAQ); K1(iTRAQ);	1402.75	42.52	55.80
												ASYLDCIR	N-Term(iTRAQ); C6(Methy)	1130.55	42.12	48.57
												DSGFQMNQLR	N-Term(iTRAQ)	1339.66	41.10	39.65
												KCSTSSLLEACTFR	N-Term(iTRAQ); K1(iTRAQ); C2(Methy); C11(Methy)	1925.92	41.09	19.64
												YLGEEYVK	N-Term(iTRAQ)	1144.60	39.47	39.25
												GDVAFVK	N-Term(iTRAQ); K7(iTRAQ)	1023.61	38.79	53.83
												HQTVPQNTGGK	K11(iTRAQ)	1310.70	37.36	44.75
												MDAKMYLGYEYVTAIR	N-Term(iTRAQ)	2068.03	36.57	43.56
												CLKDGAGDVAFVK	N-Term(iTRAQ); C1(Methy);	1656.87	35.92	51.11
												CDEWSVNSVGK	C1(Methy); K11(iTRAQ)	1413.63	35.36	42.17
												SASDLTWDNLK	N-Term(iTRAQ)	1393.71	35.26	39.60
												KSASDLTWDNLK	N-Term(iTRAQ); K12(iTRAQ)	1665.91	34.75	45.28
												SKEFQLFSSPHGK	N-Term(iTRAQ); K2(iTRAQ); K13(iTRAQ)	1924.06	32.75	23.34
												YLGEEYVKAVGNLR	N-Term(iTRAQ); K8(iTRAQ)	1899.06	32.67	29.00
												SCHTAVGR	N-Term(iTRAQ); C2(Methy)	1020.48	27.45	47.92
												EFQLFSSPHGKDLLFK	N-Term(iTRAQ); K16(iTRAQ)	2181.20	22.95	13.26
												EGTCPEAPTDECKPVK	N-Term(iTRAQ); C4(Methy); C12(Methy); K13(iTRAQ); K16(iTRAQ)	2228.04	20.95	-
												DDTVCLAK	C5(Methy); K8(iTRAQ)	1054.50	39.54	52.82
												TAGWNIPMGLLYNK	M8(Oxida); K14(iTRAQ)	1737.91	28.05	18.76
												APNHAVVTR		964.53	44.21	39.02
												KPVDEYK	N-Term(iTRAQ); K7(iTRAQ)	1166.67	42.04	-
												EGYYGYTGAFR		1283.57	38.82	39.80
												KASYLDCIR	N-Term(iTRAQ); C7(Methy)	1258.64	21.75	-
												SETKDLLFR	N-Term(iTRAQ); K4(iTRAQ)	1396.81	12.30	-
PRG2	P13727	Bone marrow proteoglycan	Down	0.45	0.53	2	2	8	12.61	136	25.2	GNLVSIHNFNINYR	N-Term(iTRAQ)	1804.96	46.55	34.27
												WNFAYWAAHQPWSR	N-Term(iTRAQ)	1963.95	38.03	35.79
CDHR2	Q9BYE9	Cadherin-related family	Down	0.47	0.51	8	8	17	7.71	524	141.5	EFYSASVAEDAAK	N-Term(iTRAQ); K13(iTRAQ)	1675.84	90.22	60.04

		member 2										DRPSLGPFLEATTTLNLFTVDQSYR	N-Term(iTRAQ)	2985.55	67.53	63.37
												LASALDYETLYTFK	N-Term(iTRAQ); K14(iTRAQ)	1923.04	55.64	-
												AWDADQTEANNR	N-Term(iTRAQ)	1534.70	50.84	51.29
												EMLVIVEDR	N-Term(iTRAQ)	1247.68	46.20	66.71
												VSALVDYER	N-Term(iTRAQ)	1195.65	43.39	47.85
												QAINAALTQATR	N-Term(iTRAQ)	1401.79	35.75	40.39
												LQFSTPK	N-Term(iTRAQ); K7(iTRAQ)	1108.66	43.12	40.12
APOA1	P02647	Apolipoprotein A-I	Down	0.48	0.52	36	36	1246	85.02	28076	30.8	LLDNWDSVTSTFSK	N-Term(iTRAQ); K14(iTRAQ)	1900.99	103.47	96.54
												DYVSQFEGSALGK	N-Term(iTRAQ); K13(iTRAQ)	1688.88	99.98	92.88
												VSFLSALEEYTK	N-Term(iTRAQ); K12(iTRAQ)	1674.92	99.62	79.54
												VSFLSALEEYTKK	N-Term(iTRAQ); K12(iTRAQ); K13(iTRAQ)	1947.12	91.46	50.65
												DLATVYVDVLK	N-Term(iTRAQ); K11(iTRAQ)	1523.89	89.71	90.72
												EQLGPVTQEFWDNLEK	N-Term(iTRAQ); K16(iTRAQ)	2221.13	87.25	86.97
												QGLLPVLESFKVSFLSALEEYTK	N-Term(iTRAQ); K11(iTRAQ);	3030.72	86.33	55.70
												QGLLPVLESFKVSFLSALEEYTK	N-Term(iTRAQ); K23(iTRAQ)	2886.62	84.94	63.73
												VKDLATVYVDVLK	N-Term(iTRAQ); K2(iTRAQ); K13(iTRAQ)	1895.16	83.43	77.72
												VKDLATVYVDVLK	N-Term(iTRAQ); K13(iTRAQ)	1751.06	82.66	90.31
												ATEHLSTLSEK	N-Term(iTRAQ); K11(iTRAQ)	1503.83	79.06	86.58
												THLAPYSDELR	N-Term(iTRAQ)	1445.75	76.61	56.58
												VQPYLDDFQK	N-Term(iTRAQ); K10(iTRAQ)	1540.83	76.04	76.88
												VSFLSALEEYTK	K12(iTRAQ)	1530.82	74.56	71.27
												LLDNWDSVTSTFSK	K14(iTRAQ)	1756.89	74.44	73.26
												DYVSQFEGSALGK	K13(iTRAQ)	1544.77	72.66	87.27
												LLDNWDSVTSTFSKLR	N-Term(iTRAQ); K14(iTRAQ)	2170.18	70.90	34.40
												DLATVYVDVLKDSGR	N-Term(iTRAQ); K11(iTRAQ)	1939.08	70.73	32.68
												EQLGPVTQEFWDNLEKETEGLR	N-Term(iTRAQ); K16(iTRAQ)	2906.48	67.79	45.45
												DYVSQFEGSALGKQLNLK	N-Term(iTRAQ); K18(iTRAQ)	2285.24	66.34	43.74
												QGLLPVLESFK	N-Term(iTRAQ); K11(iTRAQ)	1518.92	63.62	68.99
												THLAPYSDELR		1301.65	62.58	43.79
												ATEHLSTLSEK	K11(iTRAQ)	1359.72	59.74	41.20
												LHELQEK	N-Term(iTRAQ); K7(iTRAQ)	1184.69	54.56	48.10
												LAEYHAK	N-Term(iTRAQ); K7(iTRAQ)	1119.64	54.56	47.57
												LSPLGEEMR	N-Term(iTRAQ)	1175.62	54.53	56.36
												HFWQQDEPPQSPWDR	N-Term(iTRAQ)	2096.97	54.00	40.32
												LSPLGEEMR		1031.52	53.30	-
												VQPYLDDFQKK	N-Term(iTRAQ); K11(iTRAQ)	1668.92	52.96	42.02
												VQPYLDDFQKK	N-Term(iTRAQ); K10(iTRAQ); K11(iTRAQ)	1813.03	50.08	29.46
												AKVQPYLDDFQK	N-Term(iTRAQ); K12(iTRAQ)	1739.96	47.96	31.52
												DSGRDYVSQFEGSALGK	N-Term(iTRAQ); K17(iTRAQ)	2104.06	47.90	57.79
												LREQLGPVTQEFWDNLEK	N-Term(iTRAQ); K18(iTRAQ)	2490.33	47.59	67.25
												AELQEGAR	N-Term(iTRAQ)	1017.55	47.46	53.54
												LREQLGPVTQEFWDNLEK	K18(iTRAQ)	2346.22	47.20	51.04
												WQEEMELYR	N-Term(iTRAQ)	1427.68	45.93	40.03
												AKPALEDLR	N-Term(iTRAQ)	1156.68	45.77	46.28
												AKPALEDLR	N-Term(iTRAQ); K2(iTRAQ)	1300.79	45.22	40.35
												QEMSKDLEEVK	N-Term(iTRAQ); M3(Oxida); K5(iTRAQ); K11(iTRAQ)	1783.95	44.66	43.89
												KWQEEMELYR	N-Term(iTRAQ); K1(iTRAQ);	1715.87	43.51	50.52
												LEALKENGGAR	N-Term(iTRAQ); K5(iTRAQ)	1445.84	39.43	48.75
												WQEEMELYR	N-Term(iTRAQ); M5(Oxida)	1443.67	39.38	50.42
												QGLLPVLESFK	K11(iTRAQ)	1374.81	39.30	31.64
												VEPLRAELQEGAR	N-Term(iTRAQ)	1611.90	38.63	46.63
												KWQEEMELYR	N-Term(iTRAQ); M6(Oxida)	1571.77	32.85	53.16
												LHELQEKLSPLGEEMR	N-Term(iTRAQ); M15(Oxida)	2069.09	31.20	15.17

												LSPLGEEMRDR	N-Term(iTRAQ)	1446.75	22.75	35.02
												LLDNWDSVTSTFSKLR	N-Term(iTRAQ)	2026.08	19.20	37.96
												ETEGLRQEMSK	N-Term(iTRAQ); M9(Oxida); K11(iTRAQ)	1611.83	41.92	29.20
												LSPLGEEMR	N-Term(iTRAQ); M8(Oxida)	1191.61	32.06	44.56
												DLEEVK	N-Term(iTRAQ); K6(iTRAQ)	1020.58	34.91	33.48
												LSPLGEEMRDR	N-Term(iTRAQ); M8(Oxida)	1462.75	30.65	-
												LHELQEK	K7(iTRAQ)	1040.59	38.37	-
												LEALKENGGAR	N-Term(iTRAQ)	1301.73	-	34.61
												QKVEPLR	N-Term(iTRAQ); K2(iTRAQ)	1157.73	-	33.80
PZP	P20742	Pregnancy zone protein	Down	0.50	0.58	28	39	398	34.14	8023	163.8	QQNAQGGFSSTQDTVVALHALSR	N-Term(iTRAQ)	2559.31	98.15	92.30
												AGAFCLSEDAGLGISSTASLR	N-Term(iTRAQ); C5(Methy)	2216.06	95.36	80.38
												AVDQSVLLMKPEAELSVSSVYNLLTVK	N-Term(iTRAQ); M9(Oxida); K10(iTRAQ); K27(iTRAQ)	3381.89	95.32	27.29
												ALLAYAFSLLGK	N-Term(iTRAQ); K12(iTRAQ)	1554.96	90.53	70.45
												LEAGINQLSFPLSSEPIQGSYR	N-Term(iTRAQ)	2550.33	74.73	20.36
												ASPAFLASQNTK	N-Term(iTRAQ); K12(iTRAQ)	1522.85	74.22	80.03
												SSGSLLNNAIK	N-Term(iTRAQ); K11(iTRAQ)	1391.81	74.22	65.67
												NQGNTWLTAFVLKTFAQAR	N-Term(iTRAQ)	2310.25	72.84	41.64
												SLFTDLVAEK	N-Term(iTRAQ); K10(iTRAQ)	1410.81	72.35	53.94
												NQGNTWLTAFVLKTFAQAR	N-Term(iTRAQ); K13(iTRAQ)	2454.35	72.27	90.25
												TLLVEAEGIEQEK	N-Term(iTRAQ); K13(iTRAQ)	1746.97	69.89	63.49
												NQGNTWLTAFVLK	N-Term(iTRAQ); K13(iTRAQ)	1780.00	68.52	76.72
												GSFALSFPVESDVAPIAR	N-Term(iTRAQ)	2007.06	68.03	61.02
												IISIMDEK	N-Term(iTRAQ); K8(iTRAQ)	1236.71	66.02	40.87
												AFQPFFVELTMPYSVIR	N-Term(iTRAQ); M11(Oxida)	2205.15	61.73	42.67
												QTLSWTVTPK	N-Term(iTRAQ); K10(iTRAQ)	1448.84	60.91	47.95
												MVSGFIPLKPTVK	N-Term(iTRAQ); M1(Oxida); K9(iTRAQ); K13(iTRAQ)	1865.13	58.37	42.76
												SYIFIDEAHITQSLTWLSQMQK	N-Term(iTRAQ); K22(iTRAQ)	2927.53	57.95	53.50
												MLQITNTGFEMK	N-Term(iTRAQ); K12(iTRAQ)	1700.89	56.89	43.00
												ATVLNYLPK	N-Term(iTRAQ); K9(iTRAQ)	1306.79	55.67	65.77
												AFQPFFVELTMPYSVIR	N-Term(iTRAQ)	2189.16	54.41	38.29
												HQDGSYSTFGER	N-Term(iTRAQ)	1527.70	53.17	46.53
												IREEGTDLEVTANR	N-Term(iTRAQ)	1746.91	52.57	46.43
												IQHPFTVEEFVLPK	N-Term(iTRAQ); K14(iTRAQ)	1972.12	50.04	52.46
												QGIPFFAQVLLVDGK	N-Term(iTRAQ); K15(iTRAQ)	1920.12	49.14	57.22
												VVSVDENFRPR	N-Term(iTRAQ)	1461.80	45.22	47.98
												ATVLNYLPKCIR	N-Term(iTRAQ); C10(Methy)	1580.88	45.12	36.35
												MVSGFIPLKPTVK	N-Term(iTRAQ); M1(Oxida); K13(iTRAQ)	1721.03	44.36	19.08
												NALFCLESAWNVAK	N-Term(iTRAQ); C5(Methy);	1899.97	42.28	34.56
												NELIPLIYLENPR	N-Term(iTRAQ)	1727.98	41.79	26.23
												GVPIPNK	N-Term(iTRAQ); K7(iTRAQ)	1012.64	40.87	49.95
												QAMGELSELSFHYLIMAK	N-Term(iTRAQ); M3(Oxida); M16(Oxida); K18(iTRAQ)	2388.22	40.60	30.15
												VDLSFSPAQSPPASHAHLQVAAAPQSLCALR	N-Term(iTRAQ); C28(Methy)	3359.71	40.56	12.34
												YNILPEK	N-Term(iTRAQ); K7(iTRAQ)	1164.69	38.24	29.58
												DLFHCVSFTLPR	N-Term(iTRAQ); C5(Methy)	1624.81	35.34	36.05
												NQGNTWLTAFVLK	K13(iTRAQ)	1635.90	32.80	19.79
												YNILPEKEDSPFALK	N-Term(iTRAQ); K7(iTRAQ); K15(iTRAQ)	2196.23	32.14	24.53
												EILNSLDKEAVK	N-Term(iTRAQ); K8(iTRAQ); K12(iTRAQ)	1791.06	27.48	28.24
												YGAATFTR	N-Term(iTRAQ)	1030.55	26.89	40.59

												AVDQSVLLMKPEAELSVSSVYN LLTVK	N-Term(iTRAQ); K10(iTRAQ); K27(iTRAQ)	3365.90	10.38	-
												LPSNVVK	N-Term(iTRAQ); K7(iTRAQ)	1044.67	51.26	35.49
												IAQWQSLK	N-Term(iTRAQ); K8(iTRAQ)	1261.75	38.66	25.94
												SYIFIDEAHITQSLTWLSQMQK	N-Term(iTRAQ); M20(Oxida); K22(iTRAQ)	2943.52	13.72	-
												GEVFTLK	N-Term(iTRAQ); K7(iTRAQ)	1081.65	37.68	33.84
												VSVQLK	N-Term(iTRAQ); K6(iTRAQ)	961.63	44.42	41.05
PTPN6	P29350	Tyrosine-protein phosphatase non-receptor type 6	Down	0.50	0.57	4	4	6	6.39	131	67.5	SGMVQTEAQYK	N-Term(iTRAQ); K11(iTRAQ)	1529.79	64.01	57.18
												VGDQVTHIR	N-Term(iTRAQ)	1168.66	36.35	35.27
												NILPFDHSR	N-Term(iTRAQ)	1242.67	30.34	-
												LEGQRPENK	N-Term(iTRAQ); K9(iTRAQ)	1358.76	-	10.00
APOL1	O14791	Apolipoprotein L1	Down	0.50	0.58	15	15	99	54.27	2367	43.9	VSTQNLLLLLTDNEAWNGFVAA AELPR	N-Term(iTRAQ)	3099.66	87.80	43.00
												LTDVAPVSFFLVLDVVYLVYES	N-Term(iTRAQ); K23(iTRAQ)	2904.64	75.60	62.39
												VTEPISAESGEQVER	N-Term(iTRAQ)	1774.90	75.09	73.67
												SETAEELKK	N-Term(iTRAQ); K8(iTRAQ);	1466.84	70.62	52.10
												ILQADQEL	N-Term(iTRAQ)	1073.60	65.28	55.12
												WWTQAQAHDLVIK	N-Term(iTRAQ); K13(iTRAQ)	1884.04	65.20	60.29
												VNEPSILEMSR	N-Term(iTRAQ)	1418.74	61.15	37.52
												LNILNNNYK	N-Term(iTRAQ); K9(iTRAQ)	1393.81	59.08	48.53
												ALADGVQK	N-Term(iTRAQ); K8(iTRAQ)	1089.65	55.83	50.09
												VQQNVPSGTDTGDPQSKPLGD WAAGTMDPESSIFIEDAIK	N-Term(iTRAQ); K17(iTRAQ); M27(Oxida); K40(iTRAQ)	4650.28	49.47	-
												VAQELEEK	N-Term(iTRAQ); K8(iTRAQ)	1233.69	46.97	45.07
												WWTQAQAHDLVIK	K13(iTRAQ)	1739.94	42.74	18.46
												VQQNVPSGTDTGDPQSKPLGD WAAGTMDPESSIFIEDAIK	N-Term(iTRAQ); K17(iTRAQ); K40(iTRAQ)	4634.29	41.75	-
												VNEPSILEMSR	N-Term(iTRAQ); M9(Oxida)	1434.74	40.15	27.18
												ANLQSVPHASASRPR	N-Term(iTRAQ)	1734.95	38.23	30.24
												EFLGENISNFLSLAGNTYQLTR	N-Term(iTRAQ)	2631.35	38.20	87.95
												ALDNLAR	N-Term(iTRAQ)	916.54	42.93	42.23
												KALDNLAR	N-Term(iTRAQ); K1(iTRAQ)	1188.73	29.38	-
PON3	Q15166	Serum paraoxonase/lactonase 3	Down	0.51	0.54	3	3	54	7.34	1043	39.6	ILIGTVFHK	N-Term(iTRAQ); K9(iTRAQ)	1315.84	65.95	54.44
												STVEIFK	N-Term(iTRAQ); K7(iTRAQ)	1111.66	54.38	55.16
												IQNVLSEKPR	N-Term(iTRAQ); K8(iTRAQ)	1471.88	33.54	44.38
SAA1	P0DJI8	Serum amyloid A-1 protein	Down	0.52	0.60	4	11	361	77.05	11355	13.5	FFGHGAEDSLADQAANEWGR	N-Term(iTRAQ)	2322.07	126.17	118.15
												FFGHGAEDSLADQAANEWGR		2177.95	96.42	42.28
												GPGGVWAAEAISDAR		1456.72	96.22	71.91
												RGPGGVWAAEAISDAR	N-Term(iTRAQ)	1756.92	83.44	69.05
												SFFSFLGEAFDGAR		1550.74	80.21	90.13
												SFFSFLGEAFDGAR	N-Term(iTRAQ)	1694.84	76.13	76.43
												EANYIGSDK	N-Term(iTRAQ); K9(iTRAQ)	1284.67	64.82	61.07
												EANYIGSDKYFHAR	N-Term(iTRAQ); K9(iTRAQ)	1959.00	49.00	36.12
												EANYIGSDKYFHAR	K9(iTRAQ)	1814.90	48.35	47.95
												DPNHFRPAGLPEK	N-Term(iTRAQ); K13(iTRAQ)	1765.96	43.43	34.49
												EANYIGSDK	K9(iTRAQ)	1140.56	43.18	40.01
												AYSDMREANYIGSDK	N-Term(iTRAQ); M5(Oxida); K15(iTRAQ)	2023.96	38.62	27.92
												DPNHFRPAGLPEKY	N-Term(iTRAQ); K13(iTRAQ)	1929.03	32.04	48.97
												GNYDAAK	N-Term(iTRAQ); K7(iTRAQ)	1026.55	30.85	40.80
												GPGGVWAAEAISDAR	N-Term(iTRAQ)	1600.82	27.07	36.91
												SGKDPNHFRPAGLPEK	N-Term(iTRAQ); K3(iTRAQ); K16(iTRAQ)	2182.22	15.90	-
												DPNHFRPAGLPEKY	N-Term(iTRAQ)	1784.92	-	12.76
RBP4	P02753	Retinol-binding protein 4	Down	0.54	0.62	10	10	341	74.13	6971	23.0	LLNLDGTCADSYSFVFSR	N-Term(iTRAQ); C8(Methy)	2198.03	127.86	90.39
												KDPEGLFLQDNIVAEFSVDETGQ MSATAK	N-Term(iTRAQ); K1(iTRAQ); M24(Oxida); K29(iTRAQ)	3588.82	82.47	92.86

												KDPEGLFLQDNIVAEFSVDETGQMSATAK	N-Term(iTRAQ); M24(Oxida); K29(iTRAQ)	3444.70	78.25	41.53
												GNDDHWIVDTDYDTYAVQYSC	N-Term(iTRAQ); C21(Methy)	2826.18	76.40	110.65
												FSGTWYAMAK	N-Term(iTRAQ); K10(iTRAQ)	1449.75	73.18	59.08
												FSGTWYAMAK	N-Term(iTRAQ); M8(Oxida); K10(iTRAQ)	1465.74	70.57	69.77
												DPNGLPPEAQK	N-Term(iTRAQ); K11(iTRAQ)	1453.79	65.65	51.06
												LLNNWDVCADMVGTFTDTEDPAK	N-Term(iTRAQ); C8(Methy); K23(iTRAQ)	2889.34	64.62	76.75
												YWGVASFLQK	K10(iTRAQ)	1342.73	58.77	45.49
												YWGVASFLQK	N-Term(iTRAQ); K10(iTRAQ)	1486.83	58.64	61.79
												LLNNWDVCADMVGTFTDTEDPAK	N-Term(iTRAQ); C8(Methy); M11(Oxida); K23(iTRAQ)	2905.32	58.51	67.36
												LIVHNGYCDGR	N-Term(iTRAQ); C8(Methy)	1436.69	53.49	38.01
												QEELCLAR	N-Term(iTRAQ); C5(Methy)	1151.57	30.17	43.99
												QEELCLAR	C5(Methy)	1007.46	51.34	-
												VKENFDK	N-Term(iTRAQ); K7(iTRAQ)	1167.66	37.23	-
												VKENFDK	N-Term(iTRAQ); K2(iTRAQ);	1311.76	26.03	-
TUBA1B	P68363	Tubulin alpha-1B chain	Down	0.55	0.56	11	11	31	37.25	778	50.1	AFVHWYVGEGMEEGEFSEAR	N-Term(iTRAQ); M11(Oxida)	2490.11	79.94	48.87
												AFVHWYVGEGMEEGEFSEAR	N-Term(iTRAQ)	2474.11	73.85	59.27
												FDGALNVDLTEFQTNLVPYPR	N-Term(iTRAQ)	2553.32	66.90	-
												LISQIVSSITASLR	N-Term(iTRAQ)	1631.99	63.24	68.08
												VGINYQPPTVVPGGDLAK	N-Term(iTRAQ); K18(iTRAQ)	2113.19	62.35	46.56
												SIQFVDWCPTGFK	N-Term(iTRAQ); C8(Methy);	1861.93	62.06	39.49
												AVFVDLEPTVIDEVR	N-Term(iTRAQ)	1846.00	53.10	32.63
												IHFPLATYAPVISAEK	N-Term(iTRAQ); K16(iTRAQ)	2045.17	49.96	33.19
												TIGGGDDSFNTFFSETGAGK	N-Term(iTRAQ); K20(iTRAQ)	2296.10	48.03	30.27
												DVNAAIATIK	N-Term(iTRAQ); K10(iTRAQ)	1303.78	42.77	-
												LSVDYGK	N-Term(iTRAQ); K7(iTRAQ)	1069.61	50.98	33.47
												NLDIERPTYTNLNR	N-Term(iTRAQ)	1862.97	-	26.40
EEF1A1	P68104	Elongation factor 1-alpha 1	Down	0.56	0.58	5	5	12	13.42	332	50.1	EHALLAYTLGVK	N-Term(iTRAQ); K12(iTRAQ)	1602.95	82.38	64.60
												VETGVLKPGMVVTFAPVNVTTEVK	N-Term(iTRAQ); K7(iTRAQ); M10(Oxida); K24(iTRAQ)	2963.69	57.18	42.23
												EVSTYIK	N-Term(iTRAQ); K7(iTRAQ)	1127.66	53.83	-
												LPLQDVYK	N-Term(iTRAQ); K8(iTRAQ)	1263.76	42.87	43.53
												IGGIGTVPVGR	N-Term(iTRAQ)	1169.71	34.85	-
PROC	P04070	Vitamin K-dependent protein C	Down	0.57	0.64	7	7	15	23.43	396	52.0	ELNQAGQETLVTGWGYHSSR	N-Term(iTRAQ)	2377.16	72.75	84.00
												TFVLNFIK	N-Term(iTRAQ); K8(iTRAQ)	1269.78	52.82	53.51
												WELDLDIK	N-Term(iTRAQ); K8(iTRAQ)	1319.74	50.58	42.98
												LGDDLLQCHPAVK	N-Term(iTRAQ); C8(Methy);	1742.92	45.86	42.15
												EVFVHPNYSK	N-Term(iTRAQ); K10(iTRAQ)	1507.82	35.82	-
												RGDSPWQVVLLDSK	N-Term(iTRAQ); K14(iTRAQ)	1888.06	32.50	51.64
												STTDNDIALLHLAQPATLSQTIVPICLPDSGLAER	N-Term(iTRAQ); C26(Methy)	3864.00	-	35.30
APOA2	P02652	Apolipoprotein A-II	Down	0.57	0.55	9	9	471	74	8749	11.2	EPCVESLVSQYFQTVTDYGK	N-Term(iTRAQ); C3(Methy);	2627.25	97.24	79.97
												VKSPELQAEAK	N-Term(iTRAQ); K11(iTRAQ)	1487.87	83.57	31.97
												EPCVESLVSQYFQTVTDYGKDLMEK	N-Term(iTRAQ); C3(Methy); K20(iTRAQ); M23(Oxida); K25(iTRAQ)	3403.65	72.62	32.39
												EPCVESLVSQYFQTVTDYGKDLMEK	N-Term(iTRAQ); C3(Methy); K20(iTRAQ); K25(iTRAQ)	3387.66	71.95	23.57
												SKEQLTPLIK	N-Term(iTRAQ); K10(iTRAQ)	1444.90	71.29	47.42
												VKSPELQAEAK	N-Term(iTRAQ); K2(iTRAQ); K11(iTRAQ)	1631.97	69.58	62.49
												SPELQAEAK	N-Term(iTRAQ); K9(iTRAQ)	1260.71	61.77	53.63
												AGTELVNFLSYFVELGTQPATQ	N-Term(iTRAQ)	2529.31	56.91	34.57
												SKEQLTPLIK	N-Term(iTRAQ); K2(iTRAQ); K10(iTRAQ)	1589.01	54.98	59.23
												SPELQAEAK	K9(iTRAQ)	1116.60	52.94	40.73

												SPELQAEAKSYFEK	N-Term(iTRAQ); K14(iTRAQ)	1915.01	48.15	23.34
												EQLTPLIK	N-Term(iTRAQ); K8(iTRAQ)	1229.77	44.10	48.05
												SPELQAEAKSYFEK	N-Term(iTRAQ); K9(iTRAQ); K14(iTRAQ)	2059.11	34.50	18.33
												KAGTELVNFLSYFVELGTQPAT	N-Term(iTRAQ)	2657.41	31.83	-
												KAGTELVNFLSYFVELGTQPAT	N-Term(iTRAQ); K1(iTRAQ)	2801.50	31.17	23.49
THBS3	P49746	Thrombospondin-3	Down	0.57	0.46	2	2	2	2.2	41	104.1	AVTSVSGPGEHLR	N-Term(iTRAQ)	1453.79	29.48	-
												LVPNPNQK	N-Term(iTRAQ); K8(iTRAQ)	1197.72	30.40	-
IGFALS	P35858	Insulin-like growth factor-binding protein complex acid labile subunit	Down	0.58	0.60	23	23	179	52.4	4478	66.0	SFEGLGQLEVLTLDHNQLQEVK	N-Term(iTRAQ); K22(iTRAQ)	2785.50	112.13	77.56
												SLALGTFAHTPALASLGLSNNR	N-Term(iTRAQ)	2355.30	106.17	58.27
												DNGLVGIEEQSLWGLAELLELDLTSNQLTHLPHR	N-Term(iTRAQ)	3955.08	76.76	38.51
												LAELPADALGPLQR	N-Term(iTRAQ)	1607.93	76.16	68.62
												NLIAAVAPGAFLGLK	N-Term(iTRAQ); K15(iTRAQ)	1743.08	74.56	49.83
												LEDGLFEGLGSLWDLNLGWNSLAVLPDAAFR	N-Term(iTRAQ)	3532.79	70.81	-
												LAYLQPALFSGLAELR	N-Term(iTRAQ)	1906.09	68.87	54.22
												VAGLLEDTFPGLLGLR	N-Term(iTRAQ)	1815.06	68.30	69.27
												LWLEGNPWDCGCPLK	N-Term(iTRAQ); C10(Methy); C12(Methy); K15(iTRAQ)	2110.98	66.80	46.36
												DLHFLEELQLGHNR	N-Term(iTRAQ)	1864.98	63.58	75.31
												AFWLDVSHNR	N-Term(iTRAQ)	1388.72	58.20	63.98
												IRPHTFTGLSGLR	N-Term(iTRAQ)	1598.93	51.56	57.53
												TFTPQPPGLER		1242.65	49.50	-
												LEALPNSLLAPLGR	N-Term(iTRAQ)	1607.96	47.57	40.28
												DLHFLEELQLGHNR		1720.88	47.00	24.96
												NLIAAVAPGAFLGLKALR	N-Term(iTRAQ); K15(iTRAQ)	2083.31	42.26	29.49
												TFTPQPPGLER	N-Term(iTRAQ)	1386.75	40.68	41.82
												ANVFVQLPR	N-Term(iTRAQ)	1187.70	40.65	55.94
												LFQGLGK	N-Term(iTRAQ); K7(iTRAQ)	1050.66	39.80	52.64
												TFKDLHFLEELQLGHNR	N-Term(iTRAQ); K3(iTRAQ)	2385.29	39.69	47.19
												WLDLSHNR	N-Term(iTRAQ)	1184.63	38.77	34.67
												DFALQNPSAVPR	N-Term(iTRAQ)	1458.78	35.78	43.68
												ELVLAGNR	N-Term(iTRAQ)	1015.60	29.57	45.47
												LEYLLLSR	N-Term(iTRAQ)	1150.69	29.54	26.21
												LSHNAIASLRPR	N-Term(iTRAQ)	1478.87	15.76	-
												LFQGLGK	K7(iTRAQ)	906.55	46.95	-
												LSHNAIASLRPR		1334.77	17.54	-
CFHR2	P36980	Complement factor H-related protein 2	Down	0.59	0.57	5	11	111	56.67	2472	30.6	LQNNENNISCVER	C10(Methy)	1578.70	102.23	74.21
												INHGILYDEEK	N-Term(iTRAQ); K11(iTRAQ)	1618.87	101.30	79.09
												INHGILYDEEK	K11(iTRAQ)	1474.77	76.76	72.07
												ITCAEEGWSPTPK	N-Term(iTRAQ); C3(Methy);	1752.86	64.79	60.09
												LQNNENNISCVER	N-Term(iTRAQ); C10(Methy)	1722.81	62.49	46.86
												TGDIVEFVCK	N-Term(iTRAQ); C9(Methy);	1444.74	53.00	59.61
												LVYPSCEEK	N-Term(iTRAQ); C6(Methy);	1401.70	52.74	32.77
												NGQWSEPPK	N-Term(iTRAQ); K9(iTRAQ)	1330.70	50.86	48.29
												CLDPCVISQEIMEK	N-Term(iTRAQ); C1(Methy); C5(Methy); M12(Oxida); K14(iTRAQ)	2003.92	45.51	51.54
												CLDPCVISQEIMEK	N-Term(iTRAQ); C1(Methy); C5(Methy); K14(iTRAQ)	1987.92	44.74	31.56
												STISAEK	N-Term(iTRAQ); K7(iTRAQ)	1023.59	44.04	37.19
												GWSTPPK	N-Term(iTRAQ); K7(iTRAQ)	1060.60	43.96	49.64
												YKPFSQVPTGEVFYYSCEYNFVSPSK	N-Term(iTRAQ); K2(iTRAQ); C17(Methy); K26(iTRAQ)	3544.72	40.94	38.14
												LCFFPFVENGHSESSGQTHLEGDTVQIICNTGYR	N-Term(iTRAQ); C2(Methy); C29(Methy)	4021.81	13.99	-
TUBB	P07437	Tubulin beta chain	Down	0.59	0.60	14	14	38	45.95	783	49.6	ISVYYNEATGGK	N-Term(iTRAQ); K12(iTRAQ)	1589.84	78.38	59.22
												GHYTEGAELVDSVLDVVR	N-Term(iTRAQ)	2103.09	78.13	33.76

											EVDEQMLNVQNK	N-Term(iTRAQ); M6(Oxida); K12(iTRAQ)	1750.88	69.46	83.19
											LTTPTYGDLNHLVSATMSGVTTCLR	N-Term(iTRAQ); C23(Methy)	2841.41	56.92	28.51
											MAVTFIGNSTAIQELFK	N-Term(iTRAQ); K17(iTRAQ)	2158.18	56.91	42.17
											IMNTFSVVPSPK	N-Term(iTRAQ); K12(iTRAQ)	1607.91	56.39	77.15
											SGPFGQIFRPDNFVFGQSGAGNNWAK	N-Term(iTRAQ); K26(iTRAQ)	3086.56	47.52	34.90
											IMNTFSVVPSPK	N-Term(iTRAQ); M2(Oxida); K12(iTRAQ)	1623.90	43.48	44.78
											AILVDLEPGTMDSVR	N-Term(iTRAQ)	1759.94	41.49	48.23
											LHFFMPGFAPLTSR	N-Term(iTRAQ)	1764.94	35.94	28.90
											LAVNMVPFPR	N-Term(iTRAQ)	1287.74	34.85	48.04
											FPGQLNADLR	N-Term(iTRAQ)	1274.70	32.97	-
											ALTVPELTQQVFDAK	N-Term(iTRAQ); K15(iTRAQ)	1948.10	27.65	19.77
											ISEQFTAMFR	N-Term(iTRAQ); M8(Oxida)	1389.69	30.98	26.53
											IREEYPDR	N-Term(iTRAQ)	1221.64	22.61	24.16
HBD	P02042	Hemoglobin subunit delta	Down	0.60	0.54	2	8	74	52.38	1941	16.0	VLGAFSDGLAHLDNLK	N-Term(iTRAQ); K16(iTRAQ)	1958.10	95.75	97.48
											VVAGVANALAHK	N-Term(iTRAQ); K12(iTRAQ)	1437.88	83.66	86.79
											VHLTPEEK	N-Term(iTRAQ); K8(iTRAQ)	1240.72	66.39	59.73
											KVLGAFSDGLAHLDNLK	N-Term(iTRAQ); K1(iTRAQ); K17(iTRAQ)	2230.29	63.93	38.89
											VLGAFSDGLAHLDNLK	K16(iTRAQ)	1814.00	62.69	13.78
											VVAGVANALAHK	K12(iTRAQ)	1293.78	59.84	44.77
											LHVDPENFR	N-Term(iTRAQ)	1270.67	54.00	37.59
											TAVNALWGK	N-Term(iTRAQ); K9(iTRAQ)	1247.73	52.91	50.78
											EFTPQMQAAYQK	N-Term(iTRAQ); K12(iTRAQ)	1729.89	50.24	60.57
											LLVVYPWTQR	N-Term(iTRAQ)	1418.83	45.45	46.90
											VHLTPEEK	K8(iTRAQ)	1096.61	32.65	-
HNRNPU	Q00839	Heterogeneous nuclear ribonucleoprotein U	Down	0.60	0.66	2	2	4	3.76	90	90.5	EKPYFPIPEEYTFIQNVPLEDR	N-Term(iTRAQ); K2(iTRAQ)	3012.56	61.51	-
											DLPEHAVLK	N-Term(iTRAQ); K9(iTRAQ)	1309.77	25.91	-
GPLD1	P80108	Phosphatidylinositol-glycan-specific phospholipase D	Down	0.61	0.66	21	21	130	34.88	2874	92.3	QVLLVGAPTYDDVSK	N-Term(iTRAQ); K15(iTRAQ)	1893.06	95.14	55.17
											IADVTSGLIGGEDGR	N-Term(iTRAQ)	1603.84	89.15	81.14
											FGGVLHLSDLDDDGLDEIIMAAPLR	N-Term(iTRAQ)	2826.45	86.11	79.15
											TLLLVGSPTWK	N-Term(iTRAQ); K11(iTRAQ)	1502.92	77.54	65.10
											ETTLGDMTGK	N-Term(iTRAQ); K10(iTRAQ)	1340.69	77.39	62.97
											TMFIGGSQLSQK	N-Term(iTRAQ); M2(Oxida); K12(iTRAQ)	1600.86	75.78	68.96
											VYLIYGNDLGLPPVDLDLDKEAHR	N-Term(iTRAQ); K20(iTRAQ)	3013.63	72.79	53.98
											TMFIGGSQLSQK	N-Term(iTRAQ); K12(iTRAQ)	1584.87	72.34	59.24
											GIVAAFYSGPSLSDKEK	N-Term(iTRAQ); K15(iTRAQ); K17(iTRAQ)	2201.23	71.32	48.99
											FGGVLHLSDLDDDGLDEIIMAAPLR	N-Term(iTRAQ); M20(Oxida)	2842.44	69.74	45.45
											GVFFSVNSWTPDSMSFIYK	N-Term(iTRAQ); K19(iTRAQ)	2500.25	67.93	21.03
											ETTLGDMTGK	N-Term(iTRAQ); M7(Oxida); K10(iTRAQ)	1356.69	67.05	61.19
											HVSSPLASYFLSFPYAR	N-Term(iTRAQ)	2086.09	64.67	50.69
											VAFLTVTLHQGGATR	N-Term(iTRAQ)	1714.97	60.60	73.18
											AQYVLISPEASSR	N-Term(iTRAQ)	1564.85	59.02	30.94
											SWITPCPEEK	N-Term(iTRAQ); C6(Methy);	1523.75	52.49	52.39
											ELLLEHQDAYQAGIVFPDCFYPSICK	N-Term(iTRAQ); C19(Methy); C25(Methy); K26(iTRAQ)	3379.62	51.00	17.19
											LYPTYSTK	N-Term(iTRAQ); K8(iTRAQ)	1260.71	48.63	35.96
											ALEFLQLHNGR	N-Term(iTRAQ)	1441.80	46.79	42.71
											ILEGFQPSGR	N-Term(iTRAQ)	1247.69	42.97	31.38

												LGHLLHIR	N-Term(iTRAQ)	1102.70	25.86	40.50
												LSGALHVYSLGSD	N-Term(iTRAQ)	1462.76	-	61.74
												DLLGIYEK	N-Term(iTRAQ); K8(iTRAQ)	1238.72	30.33	30.93
												WYVPVK	N-Term(iTRAQ); K6(iTRAQ)	1079.65	38.45	30.28
XRCC5	P13010	X-ray repair cross-complementing protein 5	Down	0.62	0.60	2	2	4	3.01	86	82.7	SQLDIIIHSLK	N-Term(iTRAQ); K11(iTRAQ)	1554.95	39.47	42.97
												HIEIFTDLSSR	N-Term(iTRAQ)	1461.78	29.19	39.30
RPS16	P62249	40S ribosomal protein S16	Down	0.62	0.67	2	2	4	15.75	73	16.4	GGGHVAQIYAIR	N-Term(iTRAQ)	1385.78	36.60	35.52
												EIKDILIQYDR	N-Term(iTRAQ); K3(iTRAQ)	1693.98	27.30	34.96
PON1	P27169	Serum paraoxonase /arylesterase 1	Down	0.63	0.66	12	12	159	52.39	3530	39.7	ILLMDLNEEDPTVLELGITGSK	N-Term(iTRAQ); K22(iTRAQ)	2688.47	100.33	100.44
												FDVSSFNPHGISTFTDEDNAMYLLVVNHPDAK	N-Term(iTRAQ); M21(Oxida); K32(iTRAQ)	3884.87	79.90	53.53
												YVYIAELLAHK	N-Term(iTRAQ); K11(iTRAQ)	1607.94	77.14	80.45
												FDVSSFNPHGISTFTDEDNAMYLLVVNHPDAK	N-Term(iTRAQ); K32(iTRAQ)	3868.87	74.88	41.65
												IFFYDSENPPASEVLR	N-Term(iTRAQ)	2028.02	69.40	55.44
												SFNPNSPGK	N-Term(iTRAQ); K9(iTRAQ)	1235.66	64.37	53.73
												LIALTLLGMGLALFR	N-Term(iTRAQ)	1746.09	62.87	110.26
												LIALTLLGMGLALFR		1601.98	60.81	34.62
												EVQPVELPNCNLVK	N-Term(iTRAQ); C10(Methy); K14(iTRAQ)	1916.02	60.24	67.19
												IQNILTEEPK	N-Term(iTRAQ); K10(iTRAQ)	1472.86	59.20	53.22
												AKLIALTLLGMGLALFR	N-Term(iTRAQ); K2(iTRAQ); M11(Oxida)	2105.32	57.38	65.00
												SLDFNTLVDNISVDPETGDLWVGCHPNGMK	N-Term(iTRAQ); C24(Methy); K30(iTRAQ)	3607.71	48.18	10.30
												ILLMDLNEEDPTVLELGITGSK	N-Term(iTRAQ); M4(Oxida); K22(iTRAQ)	2704.48	41.59	-
												IQNILTEEPK	K10(iTRAQ)	1328.76	38.01	47.42
												VVAEGFDFANGINISPDGK	N-Term(iTRAQ); K19(iTRAQ)	2238.16	23.42	27.61
												IHVYEK	N-Term(iTRAQ); K6(iTRAQ)	1076.63	43.62	-
SAA2	P0DJI9	Serum amyloid A-2 protein	Down	0.64	0.64	2	9	213	58.2	5667	13.5	GPGGAWAAEVISNAR	N-Term(iTRAQ)	1599.84	105.58	73.98
												SFFSFLGEAFDGAR		1550.74	80.21	90.13
												SFFSFLGEAFDGAR	N-Term(iTRAQ)	1694.84	76.13	76.43
												EANYIGSDK	N-Term(iTRAQ); K9(iTRAQ)	1284.67	64.82	61.07
												RGPGGAWAAEVISNAR	N-Term(iTRAQ)	1755.94	63.45	52.69
												EANYIGSDKYFHAR	N-Term(iTRAQ); K9(iTRAQ)	1959.00	49.00	36.12
												EANYIGSDKYFHAR	K9(iTRAQ)	1814.90	48.35	47.95
												DPNHFRPAGLPEK	N-Term(iTRAQ); K13(iTRAQ)	1765.96	43.43	34.49
												EANYIGSDK	K9(iTRAQ)	1140.56	43.18	40.01
												AYSDMREANYIGSDK	N-Term(iTRAQ); M5(Oxida); K15(iTRAQ)	2023.96	38.62	27.92
												RGPGGAWAAEVISNAR		1611.84	36.74	59.85
												DPNHFRPAGLPEKY	N-Term(iTRAQ); K13(iTRAQ)	1929.03	32.04	48.97
												GNYDAAK	N-Term(iTRAQ); K7(iTRAQ)	1026.55	30.85	40.80
												DPNHFRPAGLPEKY	N-Term(iTRAQ)	1784.92	-	12.76
AFM	P43652	Afamin	Down	0.64	0.65	41	41	854	64.27	17294	69.0	DADPDTFFAK	N-Term(iTRAQ); K10(iTRAQ)	1414.71	92.44	62.78
												IAPQLSTEELVSLGEK	N-Term(iTRAQ); K16(iTRAQ)	2002.13	92.06	96.61
												ESLLNHFLYEVAR	N-Term(iTRAQ)	1734.92	86.49	75.12
												FIEDNIEYITIIAFAQYVQEATFEEMEK	N-Term(iTRAQ); K28(iTRAQ)	3672.84	85.40	62.04
												AESPEVCFNEESPK	N-Term(iTRAQ); C7(Methy);	1899.87	84.19	78.86
												HELTDEELQSLFTNFANVVDK	N-Term(iTRAQ); K21(iTRAQ)	2737.40	82.26	80.94
												RNPFVFAPTLLTVAVHFEEVAK	N-Term(iTRAQ); K22(iTRAQ)	2773.57	81.08	74.54
												TYVPPPFSQDLFTFHADMCQSQNEELQR	N-Term(iTRAQ); C19(Methy)	3518.59	76.70	32.67
												ICAMEGLPQK	N-Term(iTRAQ); C2(Methy);	1423.74	76.25	60.15
												LKHELTDEELQSLFTNFANVVD	K2(iTRAQ); K23(iTRAQ)	2978.56	74.79	32.62
												NPFVFAPTLLTVAVHFEEVAK	K21(iTRAQ)	2473.38	71.23	32.66

												NPFVFAPTLLTVAVHFEEVAK	N-Term(iTRAQ); K21(iTRAQ)	2617.46	70.90	72.35
												LPNNVLQEK	N-Term(iTRAQ); K9(iTRAQ)	1342.80	70.58	53.37
												LKHELTDEELQSLFTNFANVVD	N-Term(iTRAQ); K2(iTRAQ);	3122.68	69.63	78.55
												FTDSENVCQER	C8(Methy)	1373.55	67.33	57.37
												VVHFIYIAILSQK	N-Term(iTRAQ); K13(iTRAQ)	1819.11	66.92	80.13
												AIPVTQYLK	N-Term(iTRAQ); K9(iTRAQ)	1320.82	64.93	68.30
												HPDLSIPELLR	N-Term(iTRAQ)	1433.83	64.48	28.99
												SDVGFLPPFPTLDPEEK	N-Term(iTRAQ); K17(iTRAQ)	2176.14	63.05	42.88
												ICAMEGLPQK	C2(Methy); K10(iTRAQ)	1279.63	62.31	36.34
												HPDLSIPELLR		1289.72	61.54	25.10
												ICAMEGLPQK	N-Term(iTRAQ); C2(Methy); M4(Oxida); K10(iTRAQ)	1439.73	61.48	49.65
												IVQIYKDLLR	N-Term(iTRAQ)	1404.87	58.05	46.49
												FLVNLVK	N-Term(iTRAQ); K7(iTRAQ)	1120.74	54.60	57.18
												HFQNLGK	N-Term(iTRAQ); K7(iTRAQ)	1131.65	53.91	36.06
												TYVPPPFSQDLFTFHADMCQSQNEELQR	N-Term(iTRAQ); M18(Oxida); C19(Methy)	3534.61	53.74	25.12
												RHPDLSIPELLR	N-Term(iTRAQ)	1589.93	53.45	43.73
												RNPFVFAPTLLTVAVHFEEVAK	K22(iTRAQ)	2629.46	53.01	57.14
												KSDVGFLPPFPTLDPEEK	N-Term(iTRAQ); K18(iTRAQ)	2304.24	52.93	24.28
												LCFFYNK	N-Term(iTRAQ); C2(Methy);	1268.64	52.32	53.90
												VVHFIYIAILSQKFPK	N-Term(iTRAQ); K13(iTRAQ); K16(iTRAQ)	2335.43	50.79	47.19
												FTDSENVCQER	N-Term(iTRAQ); C8(Methy)	1517.65	50.50	52.24
												SCCEEQNK	N-Term(iTRAQ); C2(Methy); C3(Methy); K8(iTRAQ)	1320.54	49.04	39.75
												ELISLVEDVSSNYDGCCEGDVVQCIR	N-Term(iTRAQ); C16(Methy); C17(Methy); C24(Methy)	3127.34	48.90	-
												KSDVGFLPPFPTLDPEEK	N-Term(iTRAQ); K1(iTRAQ); K18(iTRAQ)	2448.34	48.46	35.24
												GQCIINSNK	N-Term(iTRAQ); C3(Methy);	1310.68	41.89	53.93
												AFSSYQK	N-Term(iTRAQ); K7(iTRAQ)	1118.61	41.76	42.36
												VVHFIYIAILSQK	K13(iTRAQ)	1675.01	41.69	21.20
												MVQQECK	N-Term(iTRAQ); C6(Methy);	1199.58	41.59	44.76
												MVQQECK	N-Term(iTRAQ); M1(Oxida); C6(Methy); K7(iTRAQ)	1215.58	39.87	40.68
												DADPDTFFAKFTFEYSR	N-Term(iTRAQ); K10(iTRAQ)	2345.13	33.27	30.10
												FIEDNIEYITIIAFAQYVQEATFEEMEK	N-Term(iTRAQ); M26(Oxida); K28(iTRAQ)	3688.81	25.65	75.80
												VVHFIYIAILSQKFPK	K13(iTRAQ); K16(iTRAQ)	2191.33	24.46	-
												RHPDLSIPELLR		1445.82	24.26	-
												CQAYESNR	N-Term(iTRAQ); C1(Methy)	1160.50	30.27	36.95
												DMVEYK	N-Term(iTRAQ); M2(Oxida);	1088.56	28.26	33.69
												YHYLIR	N-Term(iTRAQ)	1008.58	-	37.57
												IVQIYKDLLR	N-Term(iTRAQ); K6(iTRAQ)	1548.98	-	16.43
												FLVNLVKLK	N-Term(iTRAQ); K9(iTRAQ)	1361.91	37.99	23.26
												DMVEYK	N-Term(iTRAQ); K6(iTRAQ)	1072.56	34.73	37.10
												VMNHICSKQDSISSK	C6(Methy); K15(iTRAQ)	1866.91	-	33.11
												TLPECSK	N-Term(iTRAQ); C5(Methy);	1111.57	37.64	-
												IVQIYK	N-Term(iTRAQ); K6(iTRAQ)	1051.68	39.02	50.50
												FTFEYSR	N-Term(iTRAQ)	1093.55	30.45	-
												DMVEYKDR	N-Term(iTRAQ); M2(Oxida);	1359.68	26.87	26.27
												QDSISSK	K7(iTRAQ)	908.48	39.01	-
												LCFFYNK	C2(Methy); K7(iTRAQ)	1124.54	34.09	-
												VNCLQTR	N-Term(iTRAQ); C3(Methy)	1023.52	21.47	-
HBA1 PE	P69905	Hemoglobin subunit alpha	Down	0.64	0.66	6	6	93	58.45	2475	15.2	VADALTNAVAHVDDMPNALSALSDLHAHK	N-Term(iTRAQ); M15(Oxida); K29(iTRAQ)	3300.69	113.53	95.37
												VGAHAGEYGAEALER	N-Term(iTRAQ)	1673.83	84.76	80.59

			TYFPHFDLSHGSAQVK	N-Term(iTRAQ); K16(iTRAQ)	2122.09	62.45	44.35
			MFLSFPTTK	N-Term(iTRAQ); M1(Oxida);	1375.75	59.90	46.53
			MFLSFPTTK	N-Term(iTRAQ); K9(iTRAQ)	1359.76	57.41	58.41
			VDPVNFK	N-Term(iTRAQ); K7(iTRAQ)	1106.64	55.87	-
			VLSPADK	N-Term(iTRAQ); K7(iTRAQ)	1017.62	43.24	-

[a]Accession No. means accession number of protein in the UniProt/Swiss-Prot database.

[b]Protein with ratio < mean + 1SD is defined as down-regulated.

[c]The value was obtained by normalized value of CPPE patients divided by UPPE patients in experiment 1.

[d]The value was obtained by normalized value of CPPE patients divided by UPPE patients in experiment 2.

UPPE: uncomplicated parapneumonic effusion; CPPE: complicated parapneumonic effusion

Supplementary Table 4. Functional classification of 45 proteins up-regulated in CPPE compared with UPPE

Enrichment by GO Processes	Count	Genes	*p* Value
Response to bacterium	23	Leukocyte elastase, HMG1,2, MIF, Histone H2, HIST1H2BK, PGRP-S, NGAL, Fc gamma RIII alpha (CD16), PERM, Histone H2B, Alpha-defensin 1, MMP-9, ECP (RNase 3), Lysozyme, Cathepsin G, HMG2, HIST1H2BJ, BPI, Calgranulin A, Alpha-defensin, Annexin III, Calgranulin B, Azurocidin 1	2.26×10^{-20}
Defense response to bacterium	15	HMG1,2, Histone H2, HIST1H2BK, PGRP-S, PERM, Histone H2B, Alpha-defensin 1, ECP (RNase 3), Lysozyme, Cathepsin G, HIST1H2BJ, BPI, Alpha-defensin, Annexin III, Azurocidin 1	2.03×10^{-17}
Nucleosome assembly	12	Histone H4, HMG1,2, Histone H2, HIST1H2BK, Histone H2A, Histone H3.2, Histone H2B, HIST1H2BL, HMG2, HIST1H2BJ, HIST1H2AD, HIST1H2AH	3.53×10^{-16}
Defense response	24	Leukocyte elastase, HMG1,2, MIF, Histone H2, HIST1H2BK, PGRP-S, NGAL, PERM, Histone H2B, AIM (CD5L), Alpha-defensin 1, CRISP-3, ECP (RNase 3), Lysozyme, Cathepsin G, HIST1H2BJ, Annexin I, BPI, Calgranulin A, Alpha-defensin, Annexin III, Calgranulin B, Fc gamma RII alpha, Azurocidin 1	6.73×10^{-15}

The differentially expressed proteins were uploaded into MetaCore version 6.13 build 61585 (GeneGo, St. Joseph, MI) for gene ontology (GO) of cellular processes analysis.

Supplementary Table 5. Demographics and pleural fluid data of the UPPE, CPPE, and Empyema

	UPPE	CPPE	Empyema	UPPE v.s. CPPE *p*-value[b]	CPPE v.s. Empyema *p*-value[b]
Patients	35	12	21	-	-
male (%)	28 (80%)	11 (91.7%)	18 (85.7%)	-	-
Age (years)[a]	69.75 ± 3.12	66.16 ± 4.83	67.09 ± 3.93	0.994[c]	1.000[d]
Proteins (g/dl)[a]	3.64 ± 0.14	4.51 ± 0.21	3.81 ± 0.31	0.085[c]	0.347[d]
Glucose (mg/dl)[a]	154.67 ± 13.09	45.39 ± 14.58	45.14 ± 11.79	< 0.001[c]	1.000[d]
LDH (U/l)[a]	401.14 ± 52.05	1723.66 ± 429.39	8100.85 ± 2617.39	< 0.001[c]	0.001[d]
pH[a]	7.45 ± 0.02	7.21 ± 0.02	6.89 ± 0.08	0.001[c]	< 0.001[d]
BPI (ng/ml)[a]	15.9 ± 7.5	173.7 ± 51.3	332.0 ± 59.1	< 0.001[c]	0.001[d]
NGAL (ng/ml)[a]	341.4 ± 30.9	1217.6 ± 277.7	931.4 ± 135.4	< 0.001[c]	0.184[d]
AZU1 (ng/ml)[a]	248.3 ± 56.4	776.7 ± 135.0	608.3 ± 108.3	< 0.001[c]	0.464[d]
Calprotectin (ug/ml)[a]	29.8 ± 5.70	124.8 ± 13.4	168.6 ± 16.7	< 0.001[c]	0.013[d]

[a] Data are presented as mean ± s.e.m.

[b]Mann-Whitney U test.

[c]The *p* values presents the difference between uncomplicated parapneumonic effusion (UPPE) and complicated parapneumonic effusion (CPPE).

[d]The *p* values presents the difference between complicated parapneumonic effusion (CPPE) and empyema.

Supplementary Table 6. Operating characteristics of newer pleural fluid tests for identifying patients with a PPE need chest drainage.

Pleural fluid parameter	Cutoff	Sensitivity, %	Specificity, %	AUC, (95% CI)
BPI	> 7.5 ng/ml	81.4	72.0	0.793 (0.678-0.908)
NGAL	> 531 ng/ml	65.1	88.0	0.756 (0.632-0.881)
AZU1	> 171 ng/ml	86.0	68.0	0.760 (0.631-0.889)
Calprotectin	> 58.4 μg/ml	74.4	76.0	0.768 (0.649-0.888)

PPV: positive predictive values; NPV: negative predictive values; AUC: area under curve; Data are presented as % (95% confidence interval).

BPI: bactericidal permeability-increasing protein; NGAL: neutrophil gelatinase-associated lipocalin; AZU1: azurocidin.

PPE: parapneumonic effusion (refer to UPPE and CPPE).

Supplementary Table 7. Spearman correlation between pleural fluid levels of new biomarkers and biochemical parameters in all patients

Parameters	Glucose	pH	LDH	BPI	NGAL	AZU1	Calprotectin
Glucose	-	0.716*	-0.627*	-0.771*	-0.492*	-0.454*	-0.595*
pH	-	-	-0.827*	-0.830*	-0.636*	-0.587*	-0.828*
LDH	-	-	-	0.730*	0.725*	0.611*	0.922*
BPI	-	-	-	-	0.585*	0.502*	0.709*
NGAL	-	-	-	-	-	0.625*	0.736*
AZU1	-	-	-	-	-	-	0.646*
Calprotectin	-	-	-	-	-	-	-

BPI: bactericidal permeability-increasing protein; NGAL: neutrophil gelatinase-associated lipocalin; AZU1: azurocidin; LDH: lactate dehydrogenase. * $P < 0.01$

Supplementary Table 8. Pleural fluid levels of biomarkers in a new independent cohort (n = 44) with PPE

	UPPE	CPPE	*p*-value[b]	Cut-off	PPV (%)	NPV (%)	AUC, (95% CI)
Patients	26	18	-	-	-	-	-
male (%)	20 (76.9%)	12 (66.7%)	-	-	-	-	-
Age (years)[a]	66.6 ± 3.9	63.5 ± 2.6	0.322[c]	-	-	-	-
BPI (ng/ml)[a]	11.4 ± 7.9	222.3 ± 46.1	< 0.001[c]	> 10 ng/ml	88.9	92.3	0.972 (0.924-1.000)
NGAL (ng/ml)[a]	240.4 ± 74.1	1416.2 ± 272.6	< 0.001[c]	> 600 ng/ml	85.7	80.0	0.865 (0.756-0.975)
AZU1 (ng/ml)[a]	205.4 ± 47.2	753.7 ± 117.8	< 0.001[c]	> 175 ng/ml	60.9	81.0	0.848 (0.735-0.962)
Calprotectin (μg/ml)[a]	9.2 ± 1.9	118.2 ± 16.1	< 0.001[c]	> 90 μg/ml	100	72.2	0.968 (0.905-1.000)

[a] Data are presented as mean ± s.e.m.

[b] Mann-Whitney U test.

[c] The *p* values presents the difference between uncomplicated parapneumonic effusion (UPPE) and complicated parapneumonic effusion (CPPE).
